# Supplementary material for: The p97–Ataxin 3 complex regulates homeostasis of the DNA damage response E3 ubiquitin ligase RNF8
Source: EMBO J. 2019 Oct 15;38(21):e102361. doi: 10.15252/embj.2019102361 (PMC6826192; doi:10.15252/embj.2019102361)
Supplement: Supplementary file 1 — Appendix [file EMBJ-38-e102361-s001.pdf]

Appendix File.

The p97-Ataxin 3 complex regulates homeostasis of the DNA damage response E3-ubiquitin ligase RNF8

Abhay Narayan Singh[1]†, Judith Oehler[1]†[#], Ignacio Torrecilla[1], Susan Kilgas[1], Shudong Li[1], Bruno Vaz[1], Claire Guérillon[2], John Fielden[1], Esperanza Hernandez-Carralero[3,4], Elisa Cabrera[3,4], Iain D.C. Tullis[1], Mayura Meerang[5§], Paul R Barber[1], Raimundo Freire[3,4,6], Jason Parsons[7], Borivoj Vojnovic[1], Anne E. Kiltie[1], Niels Mailand[2] and Kristijan Ramadan[1]*

[1]Cancer Research UK/Medical Research Council Oxford Institute for Radiation Oncology, Department of Oncology, University of Oxford, Roosevelt Drive, Oxford, OX3 7DQ, UK, [2]Novo Nordisk Foundation Center for Protein Research, University of Copenhagen, 2200 Copenhagen, Denmark, [3]Unidad de Investigación, Hospital Universitario de Canarias and [4]Instituto de Tecnologías Biomédicas, Universidad de La Laguna, 38320 La Laguna, Tenerife, Spain, [5]Institute of Pharmacology and Toxicology-Vetsuisse Faculty, University of Zurich, Winterthurerstrasse 260, Zurich, CH-8057, Switzerland, [6]Universidad Fernando Pessoa Canarias, 35450 Santa Maria de Guia, Las Palmas, Spain, [7]Cancer Research Centre, Department of Molecular and Clinical Cancer Medicine, University of Liverpool, 200 London Road, Liverpool L3 9TA, UK.

*Correspondence: Kristijan Ramadan

E-mail: kristijan.ramadan@oncology.ox.ac.uk

†These authors contributed equally to this work.

[#]Present address: Department of Biochemistry, University of Oxford, Oxford, UK.

[§]Present address: Department of Thoracic Surgery, University Hospital Zurich, Zurich, Switzerland

Table of Contents.

Pages 3 – 4: Appendix Figure Legends.

Page 5: Appendix Figure S1.

Page 6: Appendix Figure S2.

Appendix Figure S1. Control experiments for p97, ATX3 and RNF8 inactivation.

(A) Western blot analysis showing endogenous RNF8 degradation kinetics in HeLa cells by CHX chase in comparison to DMSO control.

(B) Graph represents the quantification of (A) showing RNF8 degradation kinetics by CHX chase.

(C) Western blot analysis showing depletion efficiency of shRNF8 targeting endogenous RNF8 in U2OS cells.

(D) Western blot analysis showing the effect of p97 depletion (sip97) on the stability of ATX3 in HEK293 cells under physiological conditions and after IR (10 Gy).

(E) Western blot analysis showing the effect of p97 inhibition (DBEQ or NMS873; 10 μM for 8 h) on the stability of ATX3 in comparison to DMSO control in HEK293 cells.

(F) Western blot analysis showing the rescue of ATX3 stability after inhibition of the proteasome (MG132; 10 μM for 8 h) in HeLa cells.

(G) Western blot analysis showing the depletion efficiency of indicated siRNAs in U2OS cells.

(H) Representative IF micrographs in U2OS cells confirming the signal specificity for RNF8 antibody. (Scale bar 10μm)

(I) Quantification of endogenous RNF8 signal intensity in HeLa CRISPR-ATX3 knockout cells (Δ-ATX3) at UV-A micro-laser-induced DNA damage tracks 30 min and 5 h after damage induction. Graph represents the average intensity of RNF8 signal (ns $P>0.05$, ***$P<0.001$; unpaired t-test, n=2, +SEM, at least 100 nuclei per condition and experiment).

(J) Quantification of Flag-RNF8 signal intensity in HeLa CRISPR-ATX3 knockout cells at UV-A micro-laser-induced DNA damage tracks 30 min after damage induction. Graph

represents the average intensity of RNF8 signal (**P<0.01; unpaired t-test, n=2, +SEM, at least 100 nuclei per condition and experiment).

(K) Western blot analysis showing unmodified and modified forms of endogenous RNF8 at chromatin in HeLa-WT and HeLa-ΔATX3 cells in CHX chase experiment after 10 Gy of IR treatment. (* represents unspecific band)

(L) Quantification of unmodified RNF8 band as normalised to loading control (E). Graph represents accumulation of RNF8 at chromatin in HeLa-ΔATX3 cells (ns P>0.05, *P<0.05, ***P<0.001; unpaired t-test, n=2, +SEM).

(M) Representative IF images showing endogenous RNF8 signal at UV-A micro-laser-induced DNA damage tracks under proteasome-inhibited (MG132) or DMSO control conditions. (Scale bar 10μm)

(N) Quantification of (M). Graph represents the average intensity of RNF8 signal (***P<0.001; unpaired t-test, n=3, +SEM, at least 100 nuclei per condition and experiment).

Appendix Figure S2. Cell cycle profile of ATX3-inactivated human cell lines.

(A) Cell cycle profile of U2OSΔATX3 and HEK293ΔATX3 cells as compared with controls.

(B) Western blot analysis showing knock out efficiency of U2OSΔATX3 cells.

(C) Western blot analysis showing knock out efficiency of HEK293ΔATX3 cells.

(D) Cell cycle profile of ATX3-depleted (siRNA) U2OS cells as compared with controls.

(E) Western blot analysis showing ATX3 knockdown efficiency of siRNAs in U2OS cells.

(F) Cell cycle profile of ATX3-depleted (siRNA) HEK293 cells as compared with controls.

(G) Western blot analysis showing ATX3 knockdown efficiency of siRNAs in HEK293 cells.

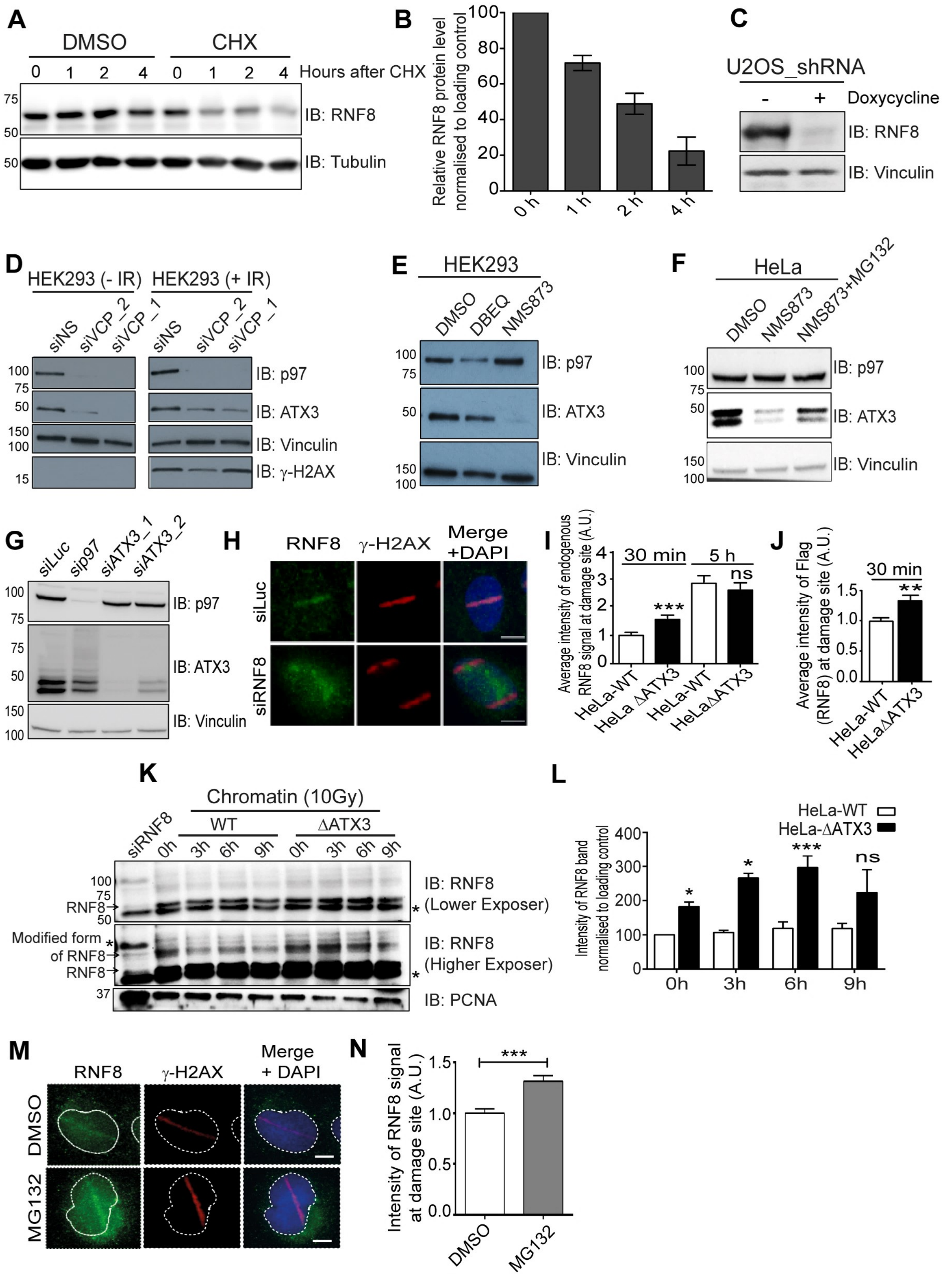
A
DMSO
CHX
0 1 2 4 0 1 2 4 Hours after CHX
IB: RNF8
IB: Tubulin
B
Relative RNF8 protein level normalised to loading control
0 h
1 h
2 h
4 h
C
U2OS_shRNA
- + Doxycycline
IB: RNF8
IB: Vinculin
D
HEK293 (- IR)
HEK293 (+ IR)
siNS
siVCP_2
siVCP_1
IB: p97
IB: ATX3
IB: Vinculin
IB: γ-H2AX
E
HEK293
DMSO
DBEQ
NMS873
IB: p97
IB: ATX3
IB: Vinculin
F
HeLa
DMSO
NMS873
NMS873+MG132
IB: p97
IB: ATX3
IB: Vinculin
G
siLuc
sip97
siATX3_1
siATX3_2
IB: p97
IB: ATX3
IB: Vinculin
H
RNF8
γ-H2AX
Merge +DAPI
siLuc
siRNF8
I
Average intensity of endogenous RNF8 signal at damage site (A.U.)
30 min
5 h
ns
HeLa-WT
HeLa ΔATX3
HeLa-WT
HeLaΔATX3
J
Average intensity of Flag (RNF8) at damage site (A.U.)
30 min
HeLa-WT
HeLaΔATX3
K
Chromatin (10Gy)
WT
ΔATX3
siRNF8
0h 3h 6h 9h 0h 3h 6h 9h
IB: RNF8 (Lower Exposer)
Modified form of RNF8
IB: RNF8 (Higher Exposer)
IB: PCNA
L
HeLa-WT
HeLa-ΔATX3
Intensity of RNF8 band normalised to loading control
0h 3h 6h 9h
ns
M
RNF8
γ-H2AX
Merge + DAPI
DMSO
MG132
N
Intensity of RNF8 signal at damage site (A.U.)
DMSO
MG132

Appendix S1

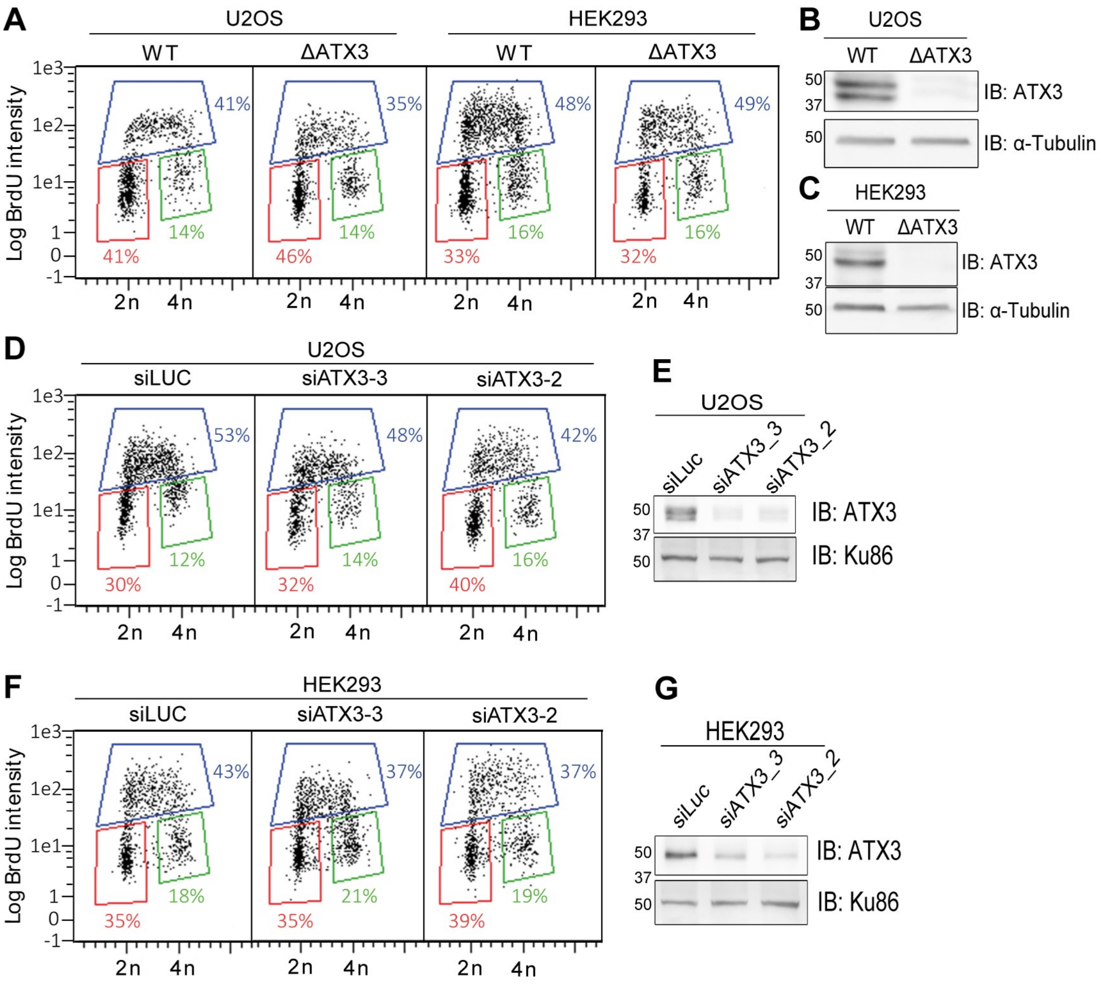
A
U2OS
HEK293
WT
ΔATX3
WT
ΔATX3
Log BrdU intensity
1e3
1e2
1e1
1
0
-1
41%
14%
41%
35%
14%
46%
48%
16%
33%
49%
16%
32%
2n 4n
B
U2OS
WT ΔATX3
50
37
IB: ATX3
50
IB: α-Tubulin
C
HEK293
WT ΔATX3
50
37
IB: ATX3
50
IB: α-Tubulin
D
U2OS
siLUC
siATX3-3
siATX3-2
53%
12%
30%
48%
14%
32%
42%
16%
40%
E
U2OS
siLuc
siATX3_3
siATX3_2
IB: ATX3
IB: Ku86
F
HEK293
siLUC
siATX3-3
siATX3-2
43%
18%
35%
37%
21%
35%
37%
19%
39%
G
HEK293
siLuc
siATX3_3
siATX3_2
IB: ATX3
IB: Ku86

Appendix S2
