# Supplementary material for: The p97–Ataxin 3 complex regulates homeostasis of the DNA damage response E3 ubiquitin ligase RNF8
Source: EMBO J. 2019 Oct 15;38(21):e102361. doi: 10.15252/embj.2019102361 (PMC6826192; doi:10.15252/embj.2019102361)
Supplement: Supplementary file 2 — Expanded View Figures PDF [file EMBJ-38-e102361-s002.pdf]

Expanded View Figures

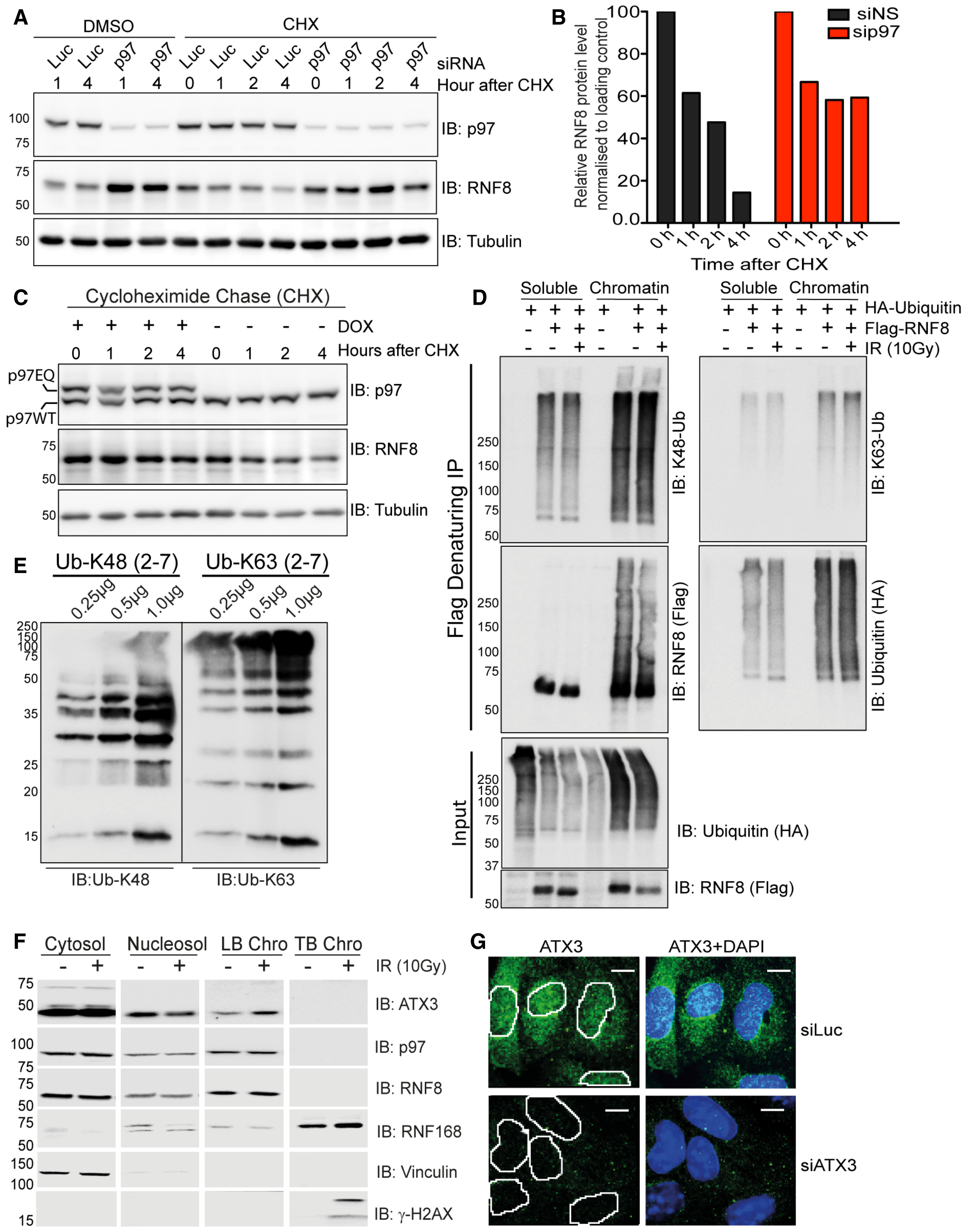

Figure EV1.

 © 2019 The Authors

Figure EV1. p97 regulates RNF8 turnover.

A Western blot analysis showing increased RNF8 protein level in HeLa cells by CHX chase after siRNA-mediated p97 depletion.
B Quantification of (A) showing reduced RNF8 degradation rate after siRNA-mediated p97 depletion.
C Western blot analysis of CHX chase in HEK293 cells showing reduced RNF8 degradation rate after doxycycline-inducible mild expression of p97EQ (dominant negative ATPase inactive) variant.
D Western blot analysis of Flag-RNF8 denaturing-IP showing RNF8 K48-Ub, K63-Ub and total-Ub (HA) ubiquitination pattern in soluble pool (cytosol + nucleosol) and on chromatin under physiological conditions and after IR (10 Gy).
E Western blot analysis showing comparison of Ub-K48 and Ub-K63 antibodies against different amounts (0.25, 0.5 and 1.0 μg) of purified recombinant K48- or K63-Ub chains (Ub-2 to Ub-7).
F Western blot analysis showing distribution of different proteins under physiological conditions and after IR (10 Gy) in cytosol, nucleosol, loosely bound chromatin (LB Chro) and tightly bound chromatin (TB Chro) of HEK293 cells.
G Representative IF images showing presence of ATX3 in the nucleus of U2OS cells (scale bar: 10 μm). Nuclei are marked by white lines.

Source data are available online for this figure.

Figure EV2. The p97–ATX3 complex regulates RNF8 turnover at sites of DNA damage.

A Western blot analysis of CHX chase kinetics showing accelerated RNF8 degradation in soluble fraction (cytoplasm + nucleoplasm) of siATX3-depleted HeLa cells.
B Graph represents the quantifications of (A) (****$P < 0.0001$; two-way ANOVA, $n = 3$, mean + SEM).
C ATX3 and RNF8 mRNA expression level analysed by quantitative PCR after indicated siRNA treatment in U2OS cells. The experiment was performed in quadruplet ($n = 1$) and columns represent the mean + SEM.
D Representative IF micrographs of U2OS cells showing recruitment of p97 or ATX3 to UV-A micro-laser-induced DNA damage tracks. Scale bar: 10 μm.
E Representative IF images showing the UV-A micro-laser-induced DNA damage tracks in U2OS cells. Endogenous RNF8 and γ-H2AX signal at damage tracks after 30 min and 5 h of damage induction under indicated siRNA-depleted conditions. Scale bar: 10 μm.
F Quantification of (E) at 30 min time point. Graph represents the average intensity of the RNF8 signal (***$P < 0.001$; unpaired t-test, $n = 3$, mean + SEM, in average, at least 70 nuclei per condition and experiment).
G Quantification of (E) at 5-h time point. Graph represents the average intensity of the RNF8 signal (*$P < 0.05$, ***$P < 0.001$; unpaired t-test, $n = 3$, mean + SEM, in average, at least 70 nuclei per condition and experiment).
H Quantification of endogenous RNF8 signal intensity in HeLa cells at UV-A micro-laser-induced DNA damage tracks 30 min and 5 h after damage induction under indicated siRNA-depleted conditions. A second, commercially available (Proteintech) RNF8 antibody was used. Graph represents the average intensity of RNF8 signal ($^{ns}P > 0.05$, **$P < 0.01$, ***$P < 0.001$; unpaired t-test, $n = 1$, mean + SEM, more than 50 nuclei were analysed per condition and experiment).
I Representative IF micrographs of HeLa cells showing Flag-RNF8 signal intensity at UV-A micro-laser-induced DNA damage tracks 30 min after damage induction under indicated siRNA-depleted conditions. Scale bar: 10 μm.
J Quantification of (I). Graph represents the average intensity of RNF8 signal (***$P < 0.001$; unpaired t-test, $n = 1$, mean + SEM, more than 50 nuclei were analysed per condition and experiment).
K Graph represents the recruitment kinetics of GFP-RNF8 at sites of two-photon laser-induced DNA damage spot in living U2OS cells under indicated conditions ($^{ns}P > 0.05$, **$P < 0.01$; unpaired t-test on area under curve, $n = 2$, for siNpl4 $n = 1$, mean + SEM, in average, at least five nuclei per condition and experiment).
L Graph represents the recruitment kinetics of GFP-RNF8 at sites of two-photon laser-induced DNA damage spot in living U2OS cells under indicated conditions (**$P < 0.01$; unpaired t-test on area under curve, $n = 3$, mean + SEM, in average, at least five nuclei per condition and experiment).
M Western blot analysis showing depletion efficiency of indicated siRNAs in U2OS cells.
N Graph represents the quantification of GFP (RNF8) foci in fixed U2OS cells after 30 min of IR (2 Gy) treatment, under indicated conditions (*$P < 0.05$, ****$P < 0.0001$; unpaired t-test, $n = 2$, +SEM).

Source data are available online for this figure.

© 2019 The Authors

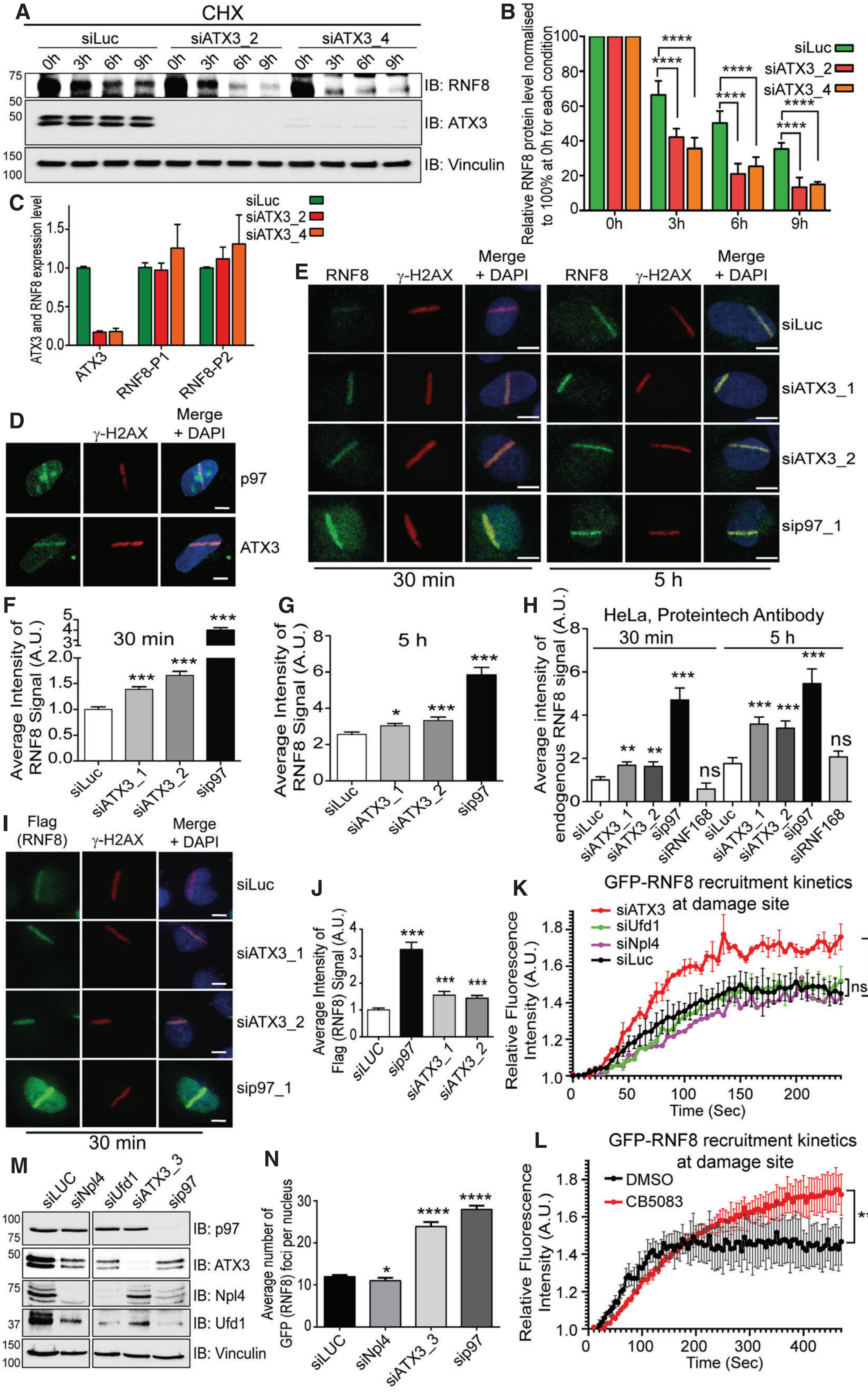

Figure EV2.

 © 2019 The Authors

Figure EV3. ATX3 operates downstream of RNF8 and K63-Ub at sites of DNA damage.

A Western blot analysis of GFP-ATX3 Co-IP in HEK293 cells showing interaction of ATX3 with endogenous RNF8 but not with MDC1 under physiological conditions and after IR (10 Gy).

B Representative IF images showing kinetics of endogenous MDC1 foci after 2 Gy of IR treatment in U2OS-WT and U2OS-ΔATX3 cells (scale bar: 10 μm).

C Quantification of (B). Graph representing percentage of nuclei with > 5 MDC1 foci, measured in more than 100 cells per condition per experiment ($^{ns}P > 0.05$, $^{**}P < 0.01$; unpaired *t*-test, $n = 2$, mean + SEM).

D Western blot analysis of EGFP-MDC1 denaturing-IP showing phospho-MDC1 (pSQ/TQ-MDC1; RNF8 recruitment motif) signal in HEK293 and HEK293ΔATX3 cells after 10 Gy of IR treatment.

E Representative IF images showing the UV-A micro-laser-induced DNA damage tracks in U2OS cells. Endogenous K63-Ub and γ-H2AX signal at damage tracks under indicated siRNA-depleted conditions. Scale bar: 10 μm.

F Quantification of (E). Graph represents the average intensity of K63-Ub ($^{***}P < 0.001$; unpaired *t*-test, $n = 3$, mean + SEM, at least 100 nuclei per condition and experiment).

G Representative IF images showing Flag-RNF8-WT or Flag-RNF8-RING* variant signal at UV-A micro-laser-induced DNA damage tracks in U2OS cells after 30 min and 5 h of damage induction. Scale bar: 10 μm.

H Quantification of (G). Graph represents the average intensity of the RNF8 signal ($^{*}P < 0.01$, $^{***}P < 0.001$; unpaired *t*-test, $n = 2$, mean + SEM, at least 50 nuclei per condition and experiment).

Source data are available online for this figure.

© 2019 The Authors

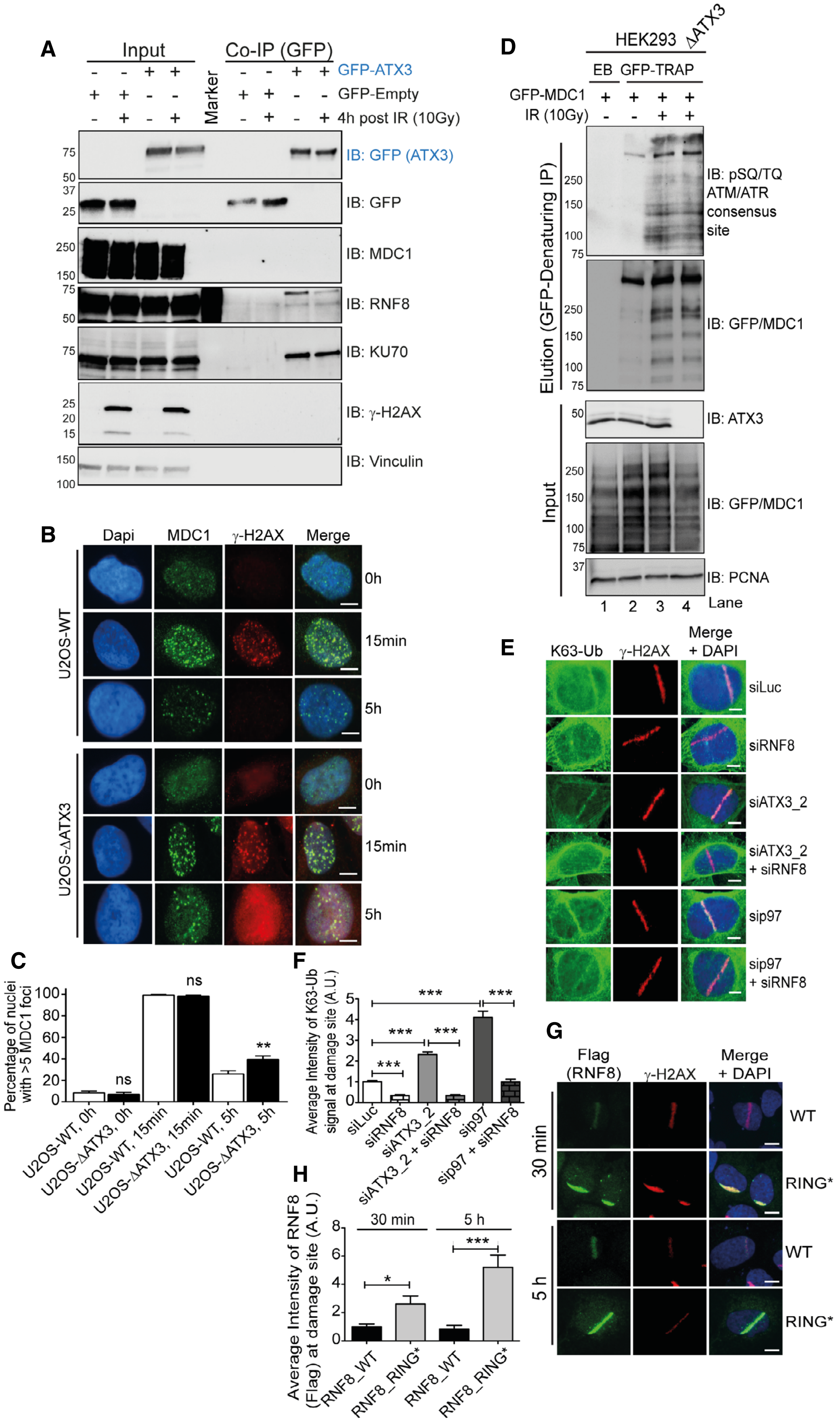

Figure EV3.

 © 2019 The Authors

Figure EV4. Separation of functions between p97 and ATX3 at sites of DNA damage.

A Representative IF images showing the UV-A micro-laser-induced DNA damage tracks in U2OS cells. Endogenous K48-Ub and γ-H2AX signal at damage tracks 30 min and 5 h after damage induction under indicated siRNA-depleted conditions. Scale bar: 10 μm.

B Quantification of (A). Graph represents the average intensity of K48-Ub signal ($^{ns}P > 0.05$, $^{**}P < 0.01$, $^{***}P < 0.001$; unpaired *t*-test, $n = 2$, mean + SEM, more than 50 nuclei were analysed per condition and experiment).

C Western blot analysis of Flag-histone H1 denaturing-IP showing ubiquitination pattern of histone H1, 30 min and 5 h post-IR (10 Gy) recovery under indicated conditions.

D Western blot analysis of Flag-histone H1 denaturing-IP showing ubiquitination pattern of histone H1, 30 min and 5 h post-IR (10 Gy) recovery under indicated conditions.

E Representative IF micrographs in U2OS cells showing IR-induced foci formation for endogenous BRCA1 and γ-H2AX under indicated siRNA-depleted conditions 2 and 6 h after IR (2 Gy) treatment. EdU click reaction was used to label S-phase cells. Scale bar: 10 μm.

F, G (F) Quantification of an average number of BRCA1 foci per EdU-positive nucleus 2 h after IR treatment, under indicated siRNA conditions. (G) Same as (F) but 6 h after IR treatment. Graphs in (F) and (G) represent the average number of BRCA1 foci per EdU-positive nucleus ($^{ns}P > 0.05$, $^{*}P < 0.05$, $^{***}P < 0.001$; unpaired *t*-test, $n = 2$, mean + SEM, at least 100 nuclei per condition and experiment).

Source data are available online for this figure.

© 2019 The Authors

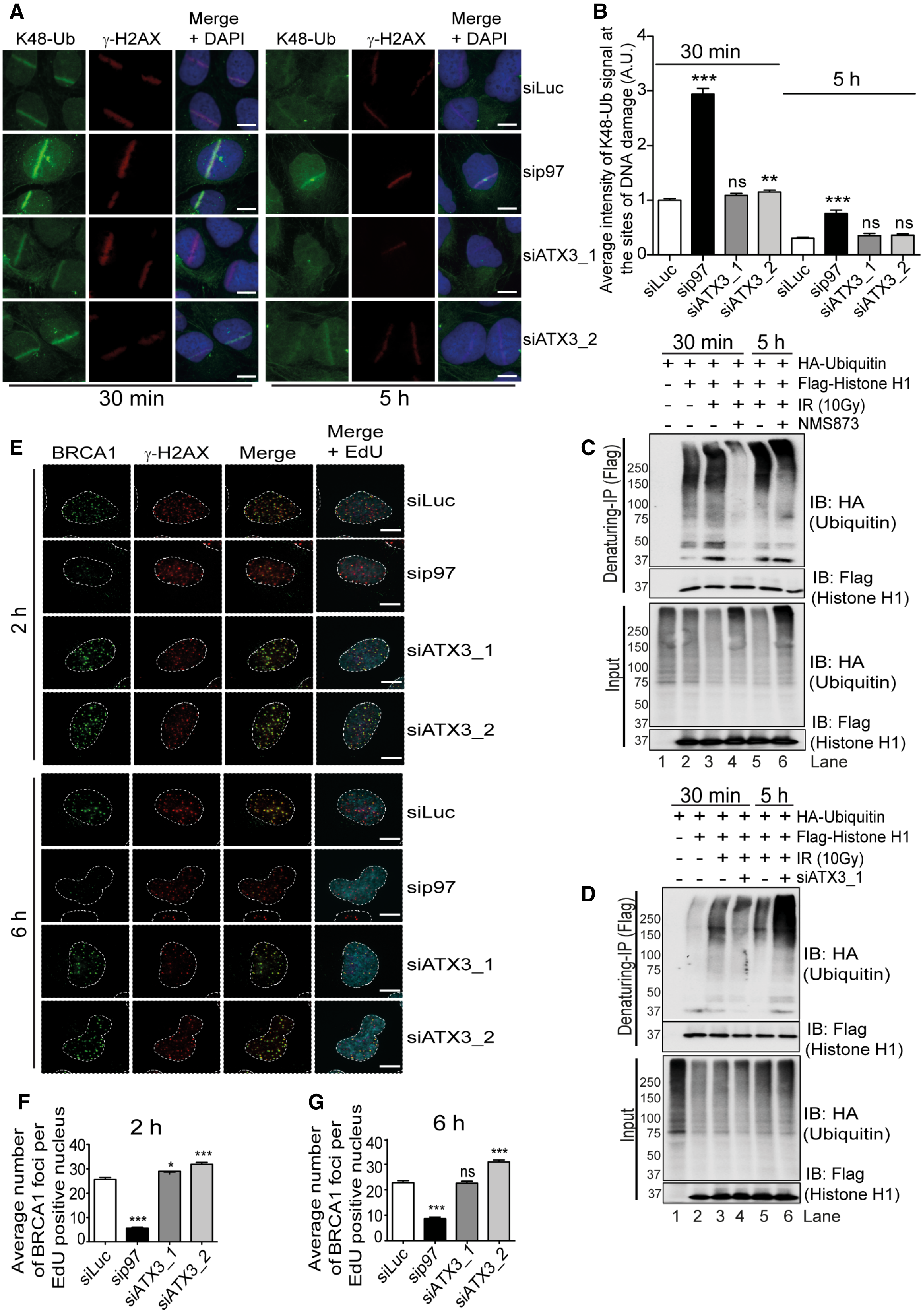

Figure EV4.

 © 2019 The Authors

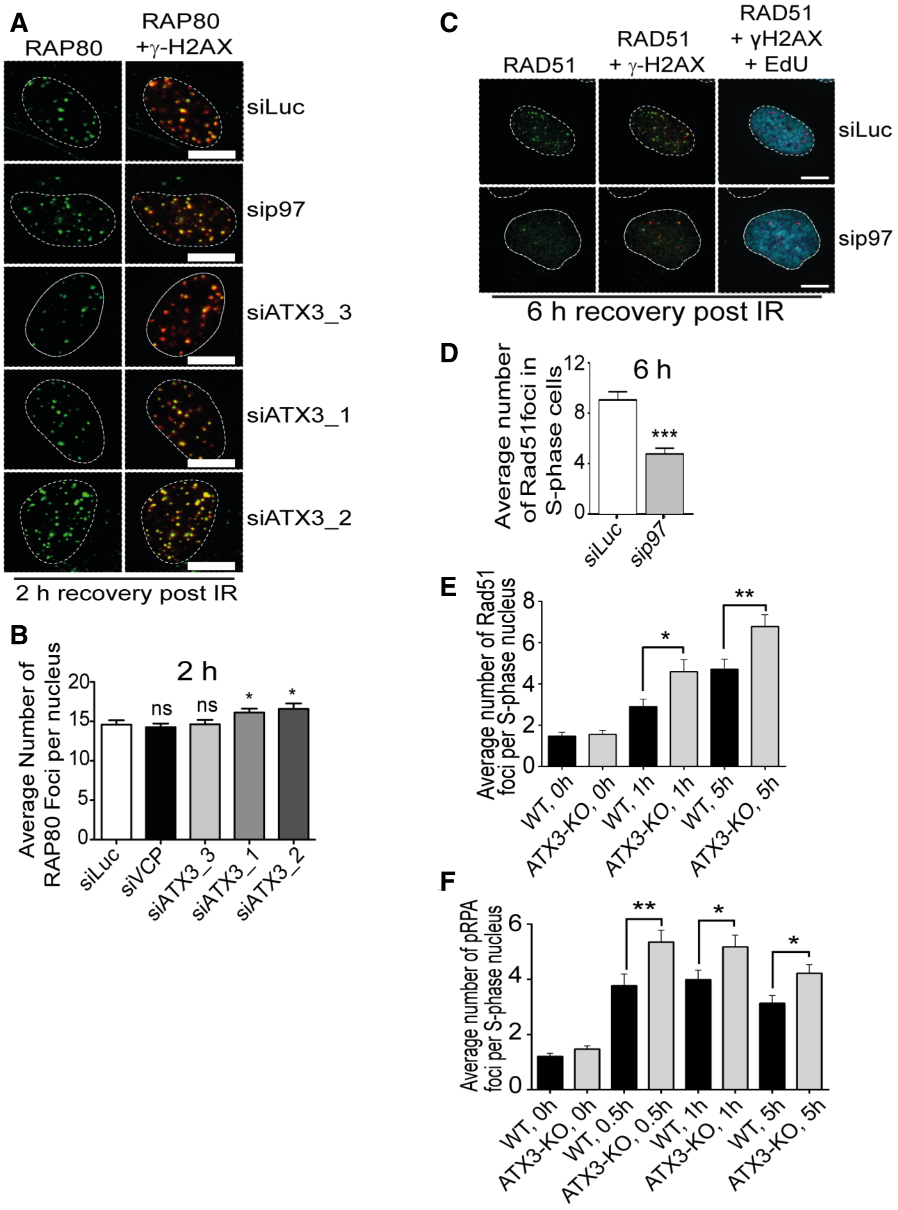

Figure EV5. ATX3 prevents excessive 5′-DNA end resection at sites of DNA damage.

A Representative IF micrographs of U2OS cells showing IR-induced foci for endogenous RAP80 and γ-H2AX under indicated siRNA-depleted conditions 2 h after IR (2 Gy) treatment. Scale bar: 10 μm.

B Quantification of (A). Graph represents the average number of RAP80 foci per nucleus ($^{ns}P > 0.05$, $*P < 0.05$; unpaired t-test, $n = 2$, mean + SEM, at least 100 nuclei per condition and experiment).

C Representative IF micrographs in U2OS cells showing IRIF for endogenous RAD51 and γ-H2AX under indicated siRNA-depleted conditions 6 h after IR (2 Gy) treatment. EdU staining was used to label S-phase cells. Scale bar: 10 μm.

D Quantification of (C). Graph represents the average number of RAD51 foci per EdU-positive nucleus ($***P < 0.001$; unpaired t-test, $n = 2$, mean + SEM, at least 100 nuclei per condition and experiment).

E Graph representing average number of Rad51 foci per S-phase (EdU-positive) nucleus in U2OSΔATX3 cells as compared with control at different time points (0, 1 and 5 h) after 2 Gy of IR treatment ($*P < 0.05$, $**P < 0.01$; unpaired t-test, $n = 3$, mean + SEM, at least 100 nuclei were counted per condition and experiment).

F Graph representing average number of pRPA foci per S-phase (EdU-positive) nucleus in U2OSΔATX3 cells as compared with control at different time points (0, 0.5, 1 and 5 h) after 2 Gy of IR treatment ($*P < 0.05$, $**P < 0.01$; unpaired t-test, $n = 3$, mean + SEM, at least 100 nuclei were counted per condition and experiment).

© 2019 The Authors
