# Supplementary material for: The p97–Ataxin 3 complex regulates homeostasis of the DNA damage response E3 ubiquitin ligase RNF8
Source: EMBO J. 2019 Oct 15;38(21):e102361. doi: 10.15252/embj.2019102361 (PMC6826192; doi:10.15252/embj.2019102361)

Figure EV1; A

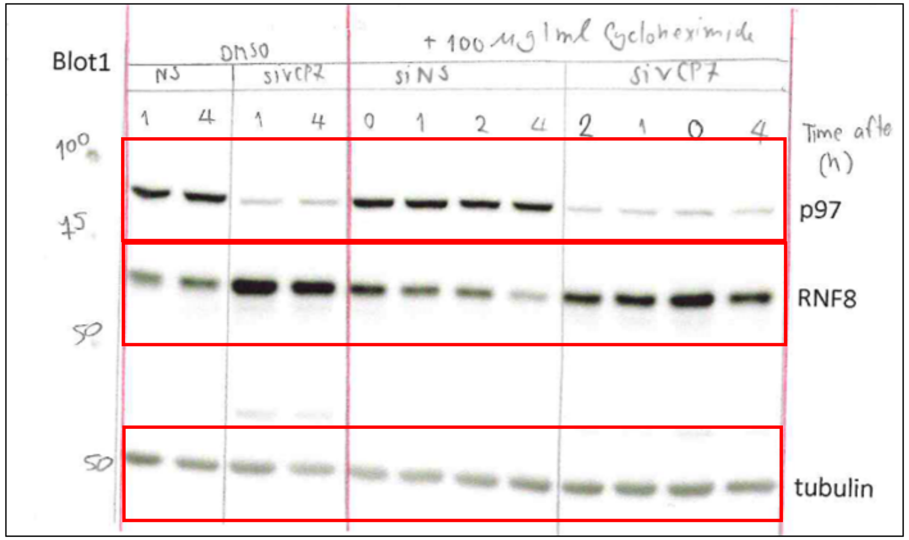

Figure EV1; C

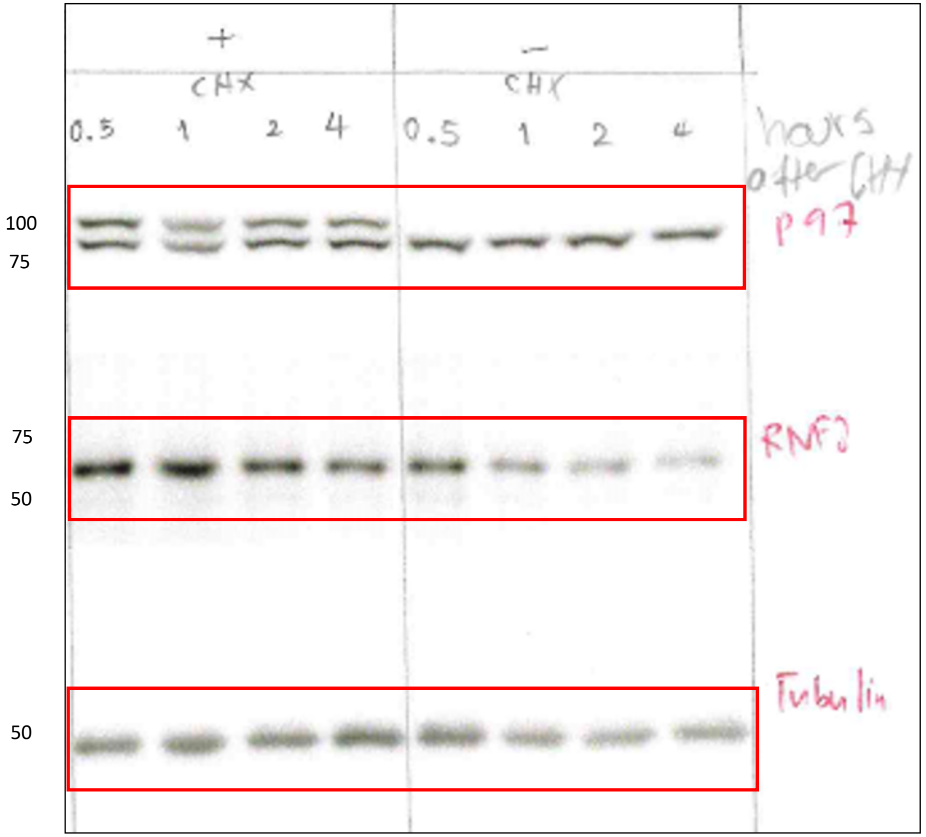

Figure EV1; D

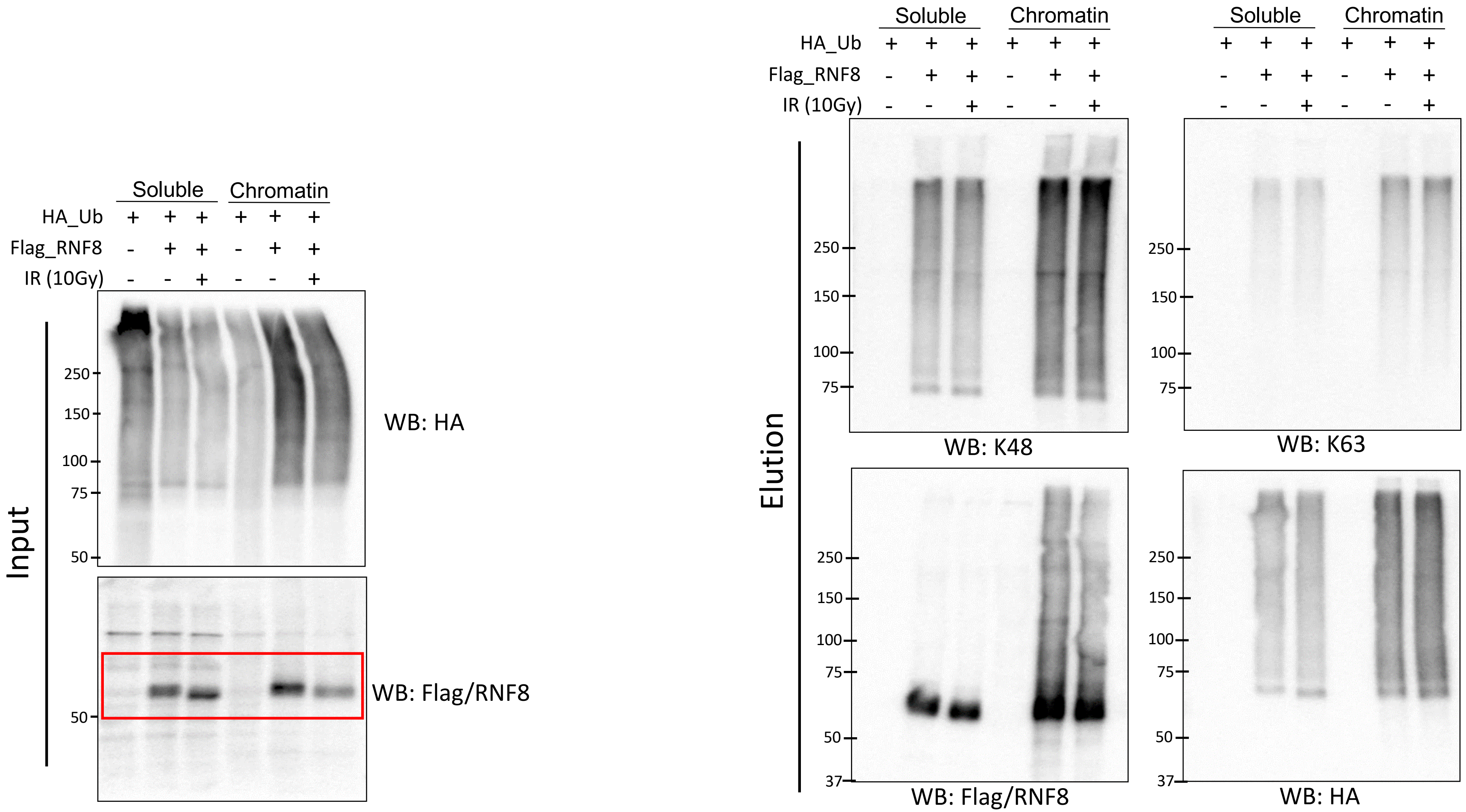

Soluble
Chromatin
HA_Ub + + + + + +
Flag_RNF8 - + + - + +
IR (10Gy) - - + - - +
Input
250
150
100
75
50
WB: HA
50
WB: Flag/RNF8
Elution
WB: K48
WB: K63
WB: Flag/RNF8
WB: HA
37

Figure EV1; E

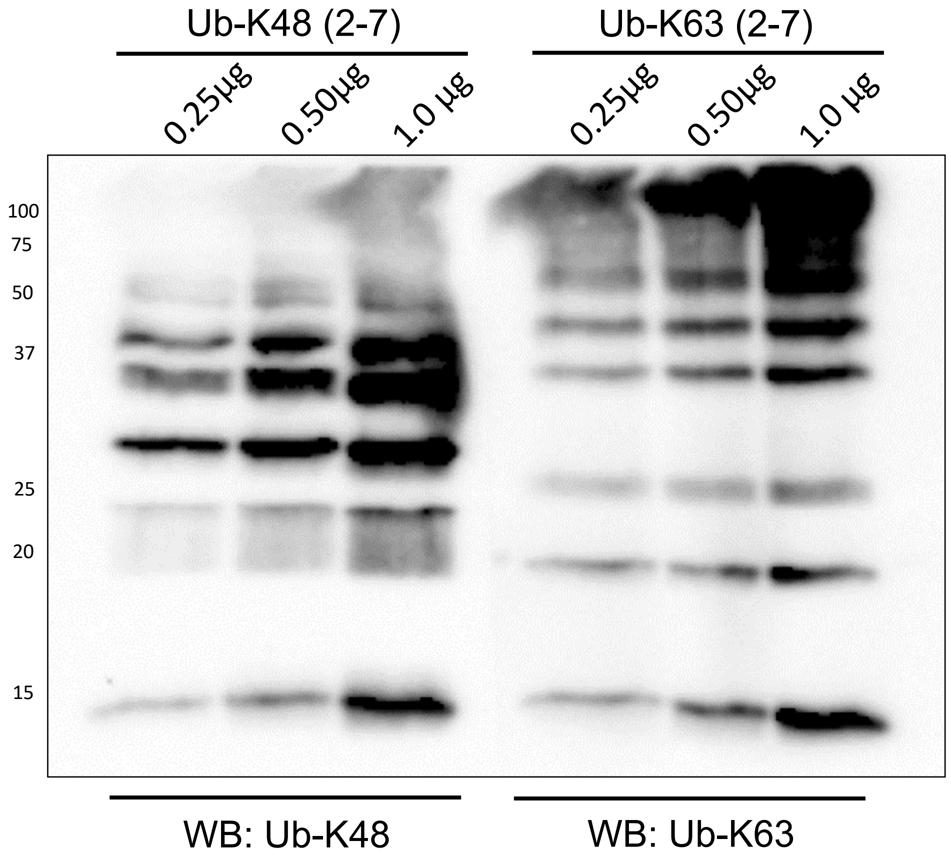

Signal only

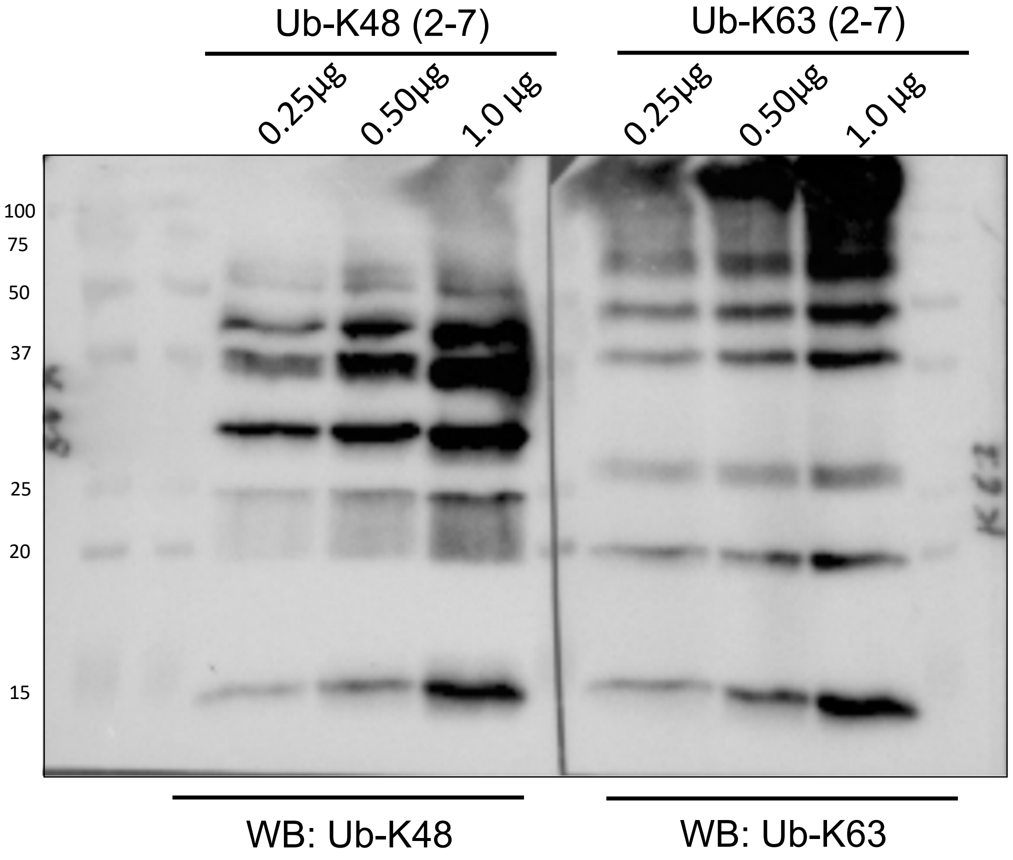

Signal superimposed on membrane

Figure EV1; F

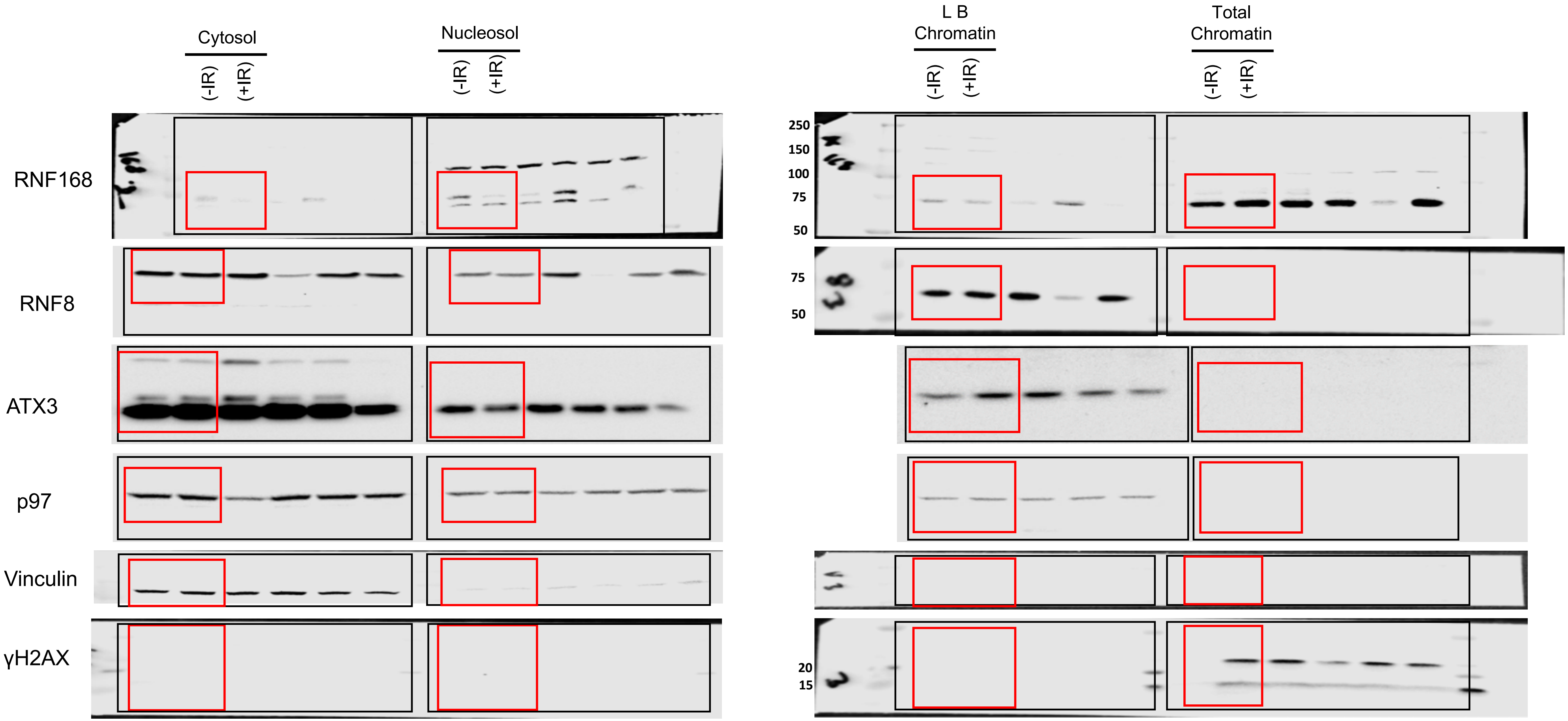

Cytosol
(-IR)
(+IR)
Nucleosol
(-IR)
(+IR)
L B Chromatin
(-IR)
(+IR)
Total Chromatin
(-IR)
(+IR)
RNF168
RNF8
ATX3
p97
Vinculin
γH2AX
250
150
100
75
50
75
50
20
15

Figure EV2; A

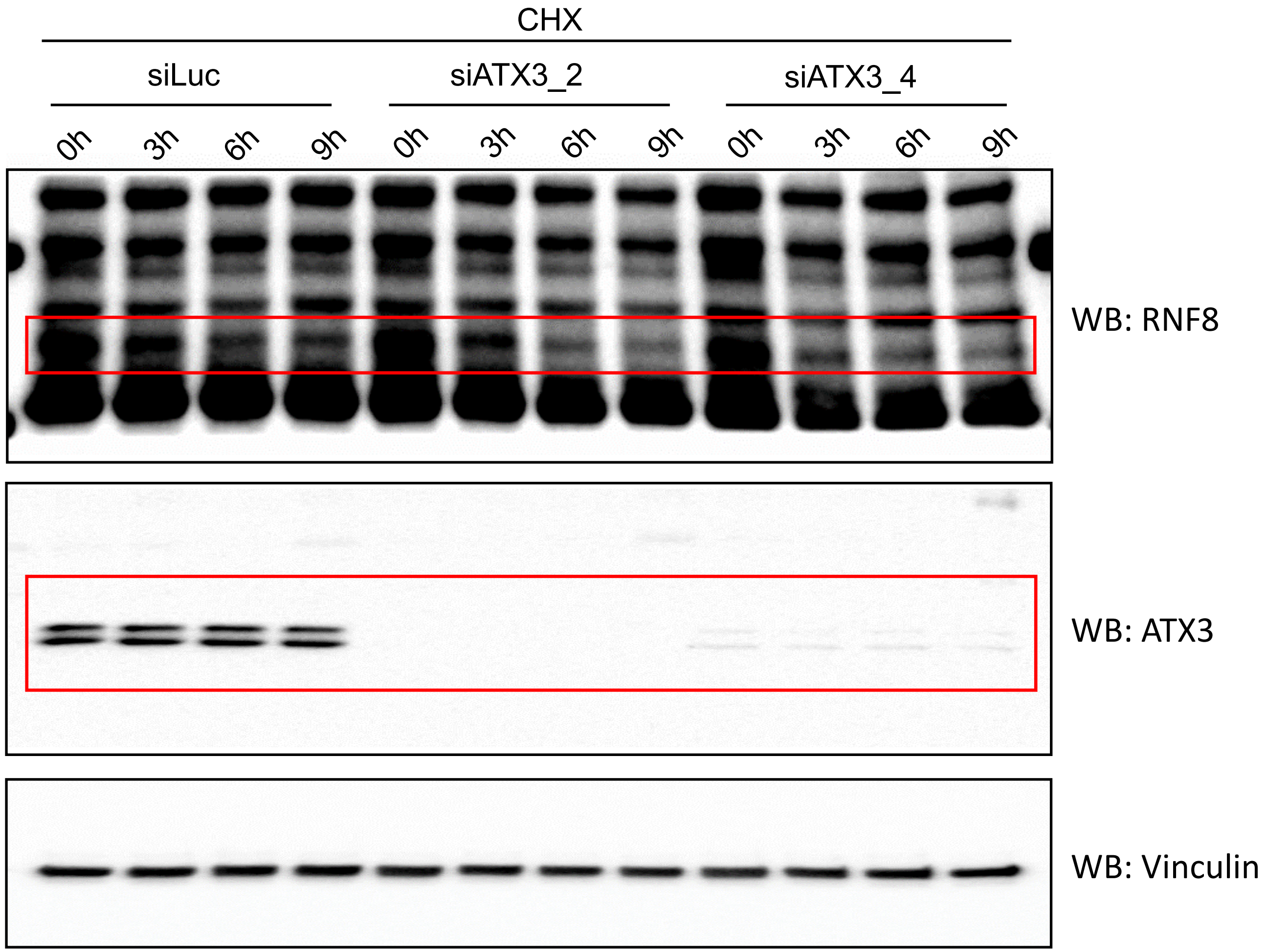

Figure EV2; M

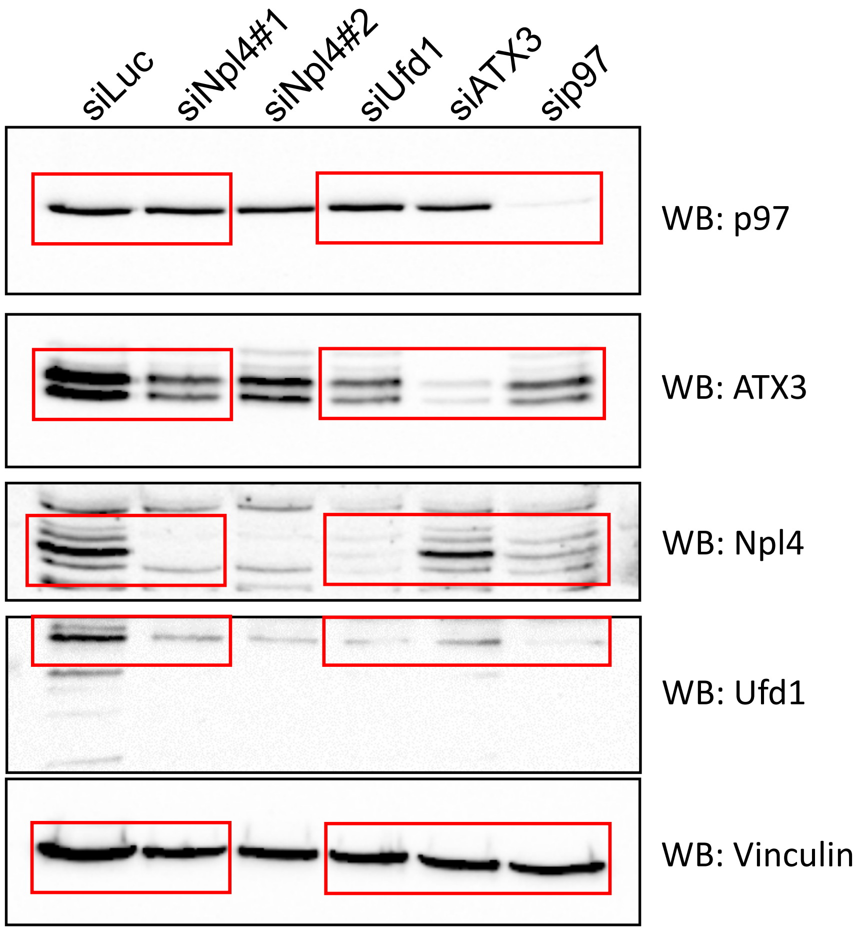

Figure EV 3 A

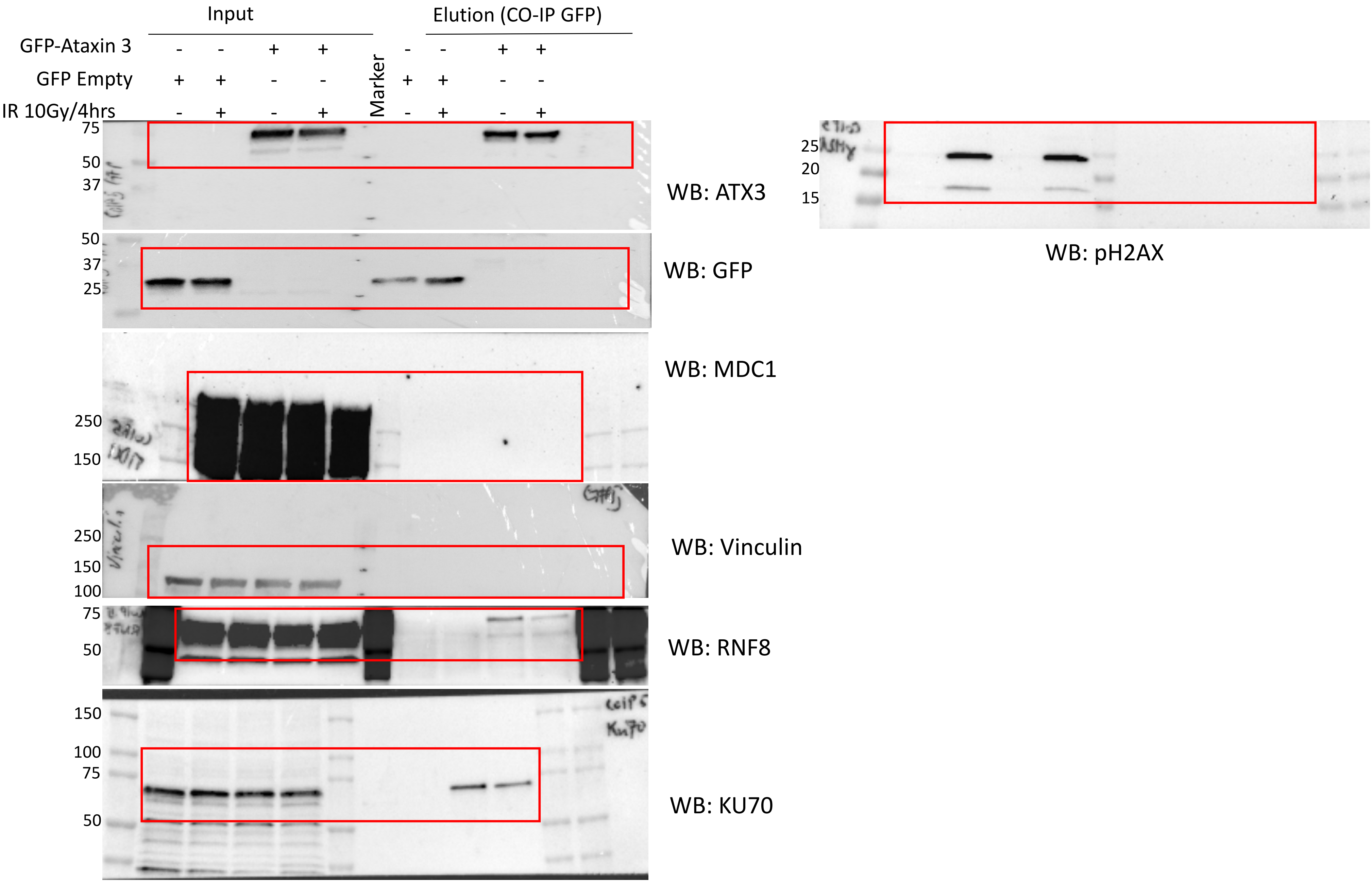
Input
Elution (CO-IP GFP)
GFP-Ataxin 3
GFP Empty
IR 10Gy/4hrs
Marker
75
50
37
WB: ATX3
50
37
25
WB: GFP
250
150
WB: MDC1
250
150
100
WB: Vinculin
75
50
WB: RNF8
150
100
75
50
WB: KU70
25
20
15
WB: pH2AX

Figure EV 3 D

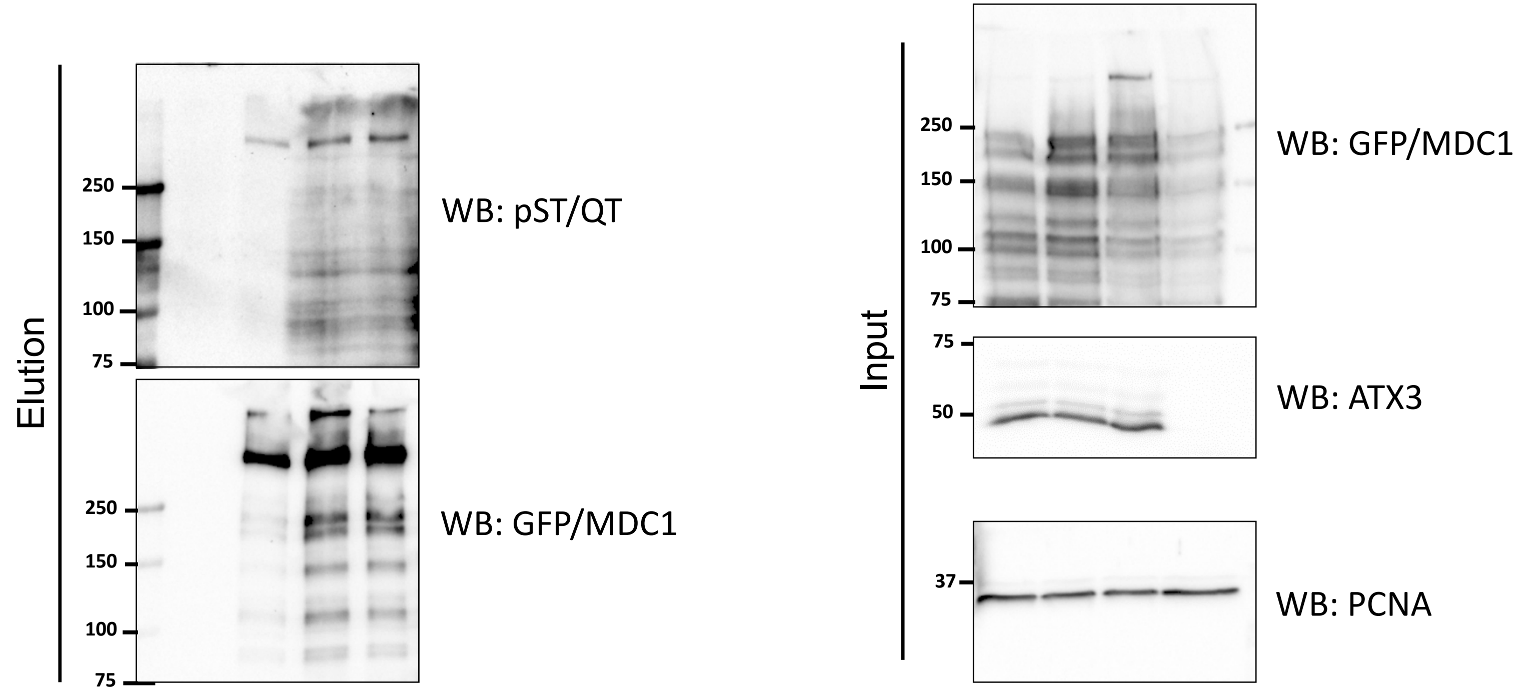
Elution
250
150
100
75
WB: pST/QT
250
150
100
75
WB: GFP/MDC1
Input
250
150
100
75
WB: GFP/MDC1
75
50
WB: ATX3
37
WB: PCNA

Figure EV4 C

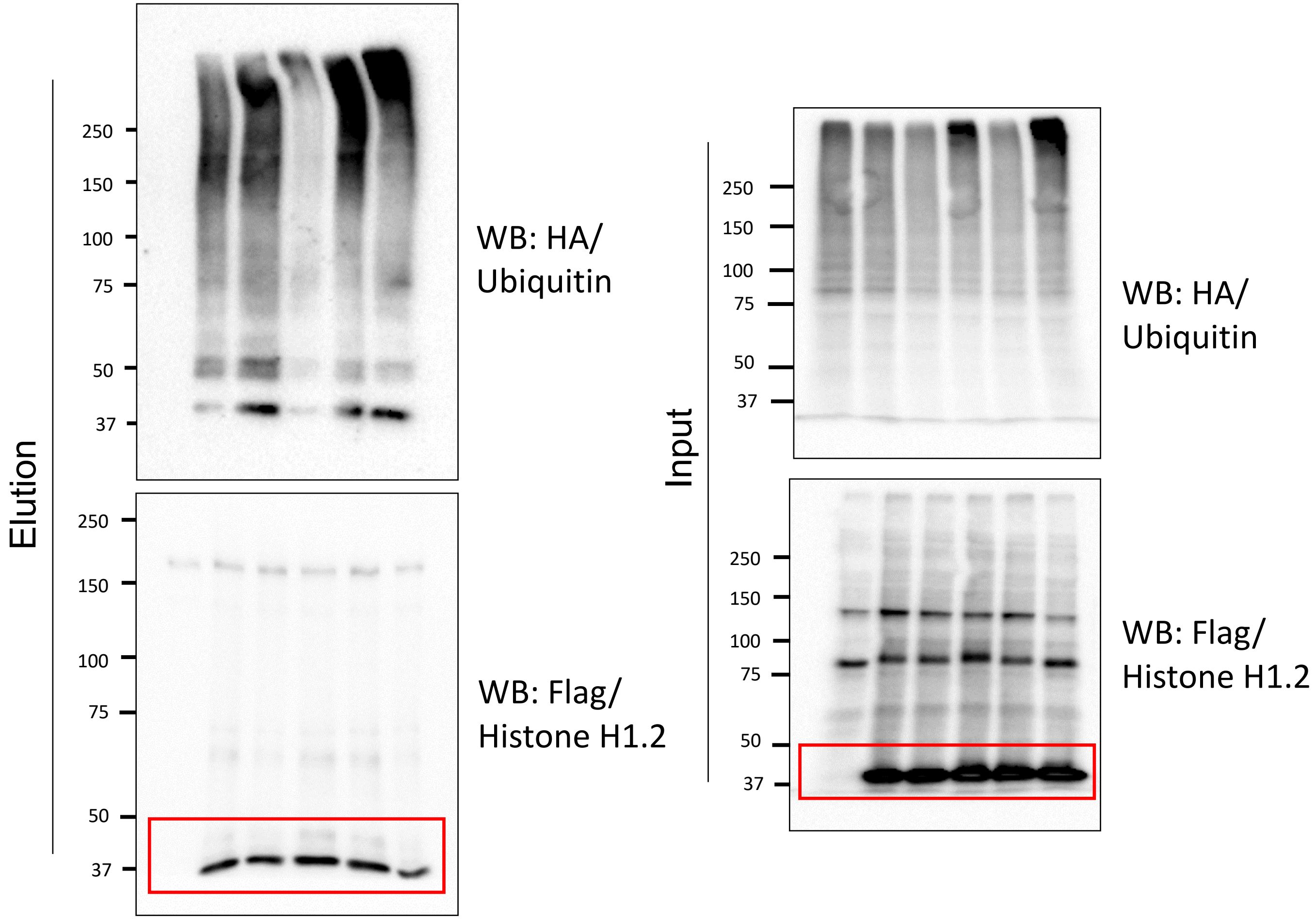

Figure EV4 D

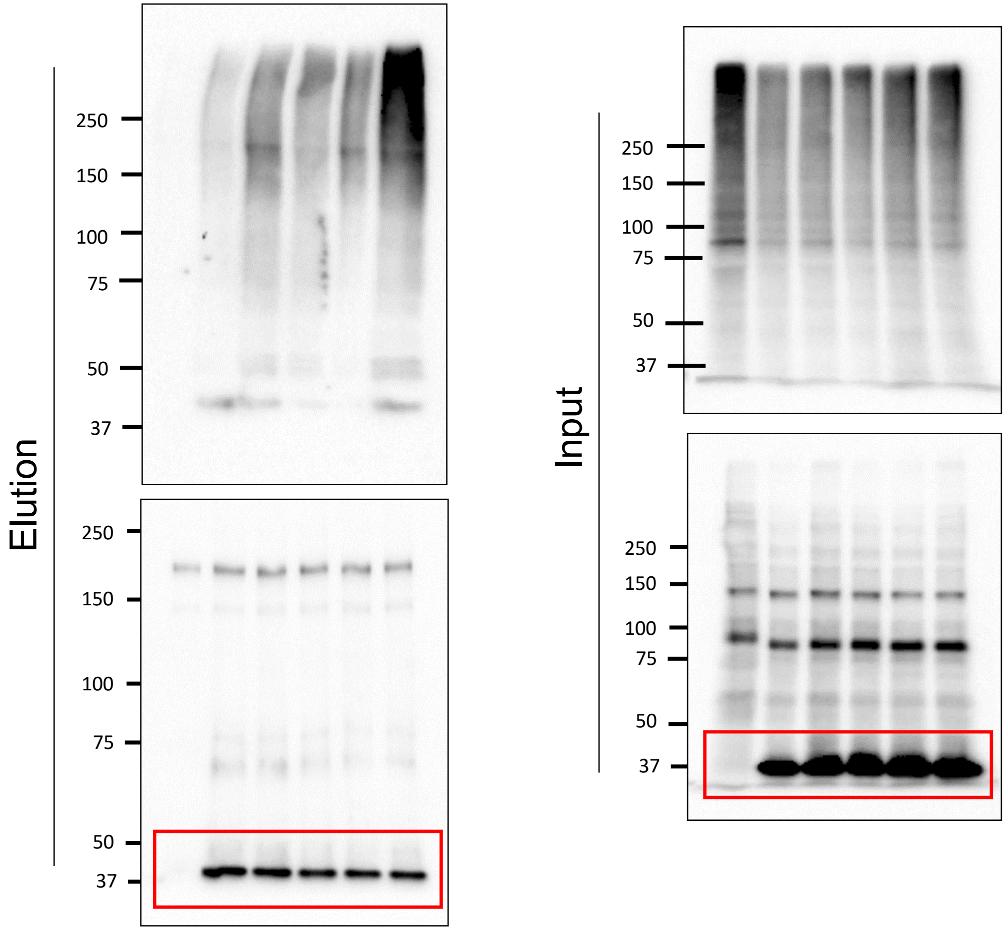

Supplement: Supplementary file 4 — Source Data for Expanded View [file EMBJ-38-e102361-s011.zip › embj2019102361-sup-0011-SDataEVFigs.pdf]
