# Supplementary figures and images for: The p97–Ataxin 3 complex regulates homeostasis of the DNA damage response E3 ubiquitin ligase RNF8
Source: EMBO J. 2019 Oct 15;38(21):e102361. doi: 10.15252/embj.2019102361 (PMC6826192; doi:10.15252/embj.2019102361)

Figure 1A

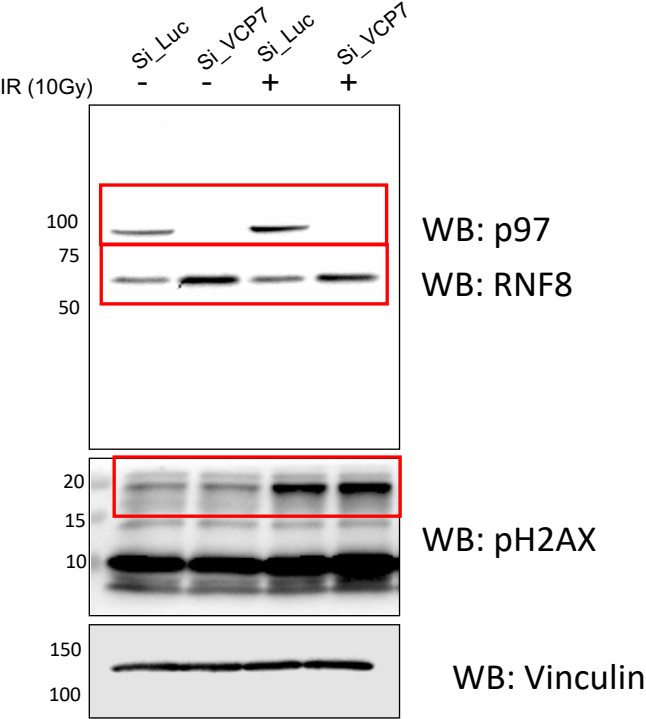

Figure 1C

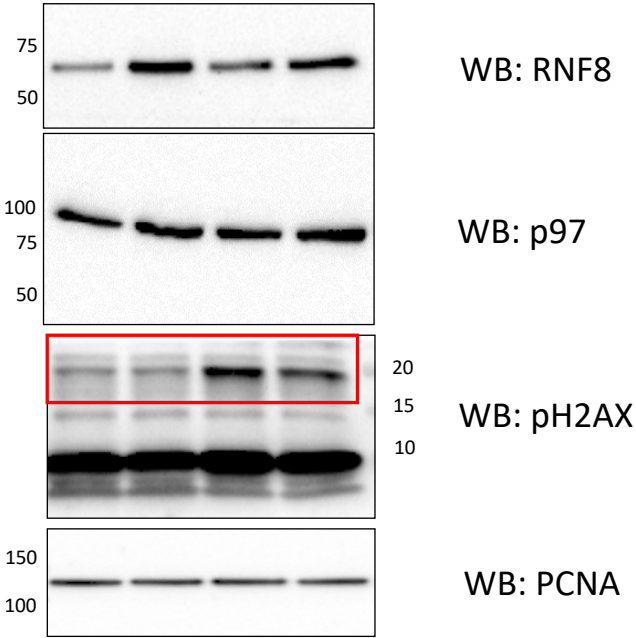

Figure 1E

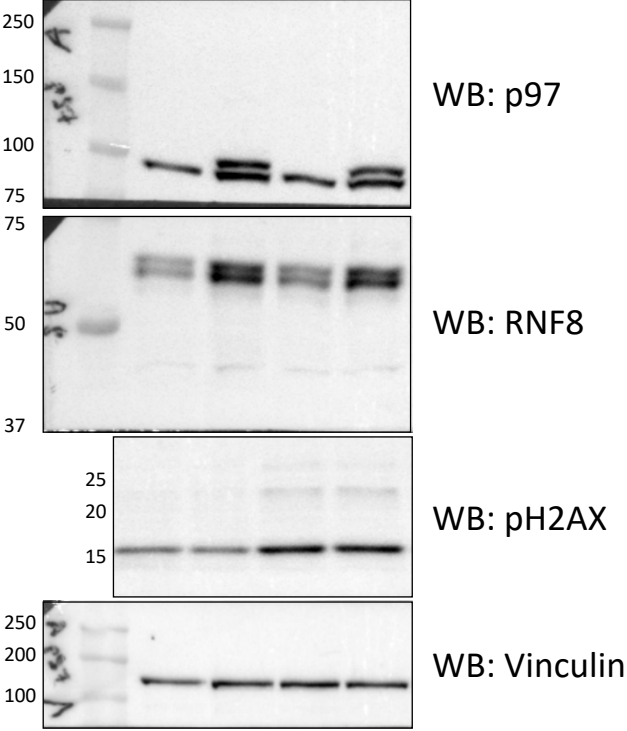

Figure 1H

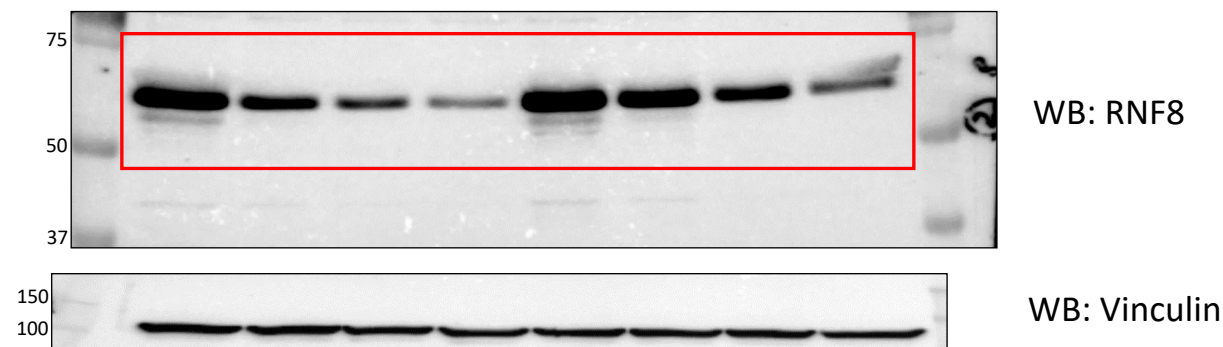

Figure 1J

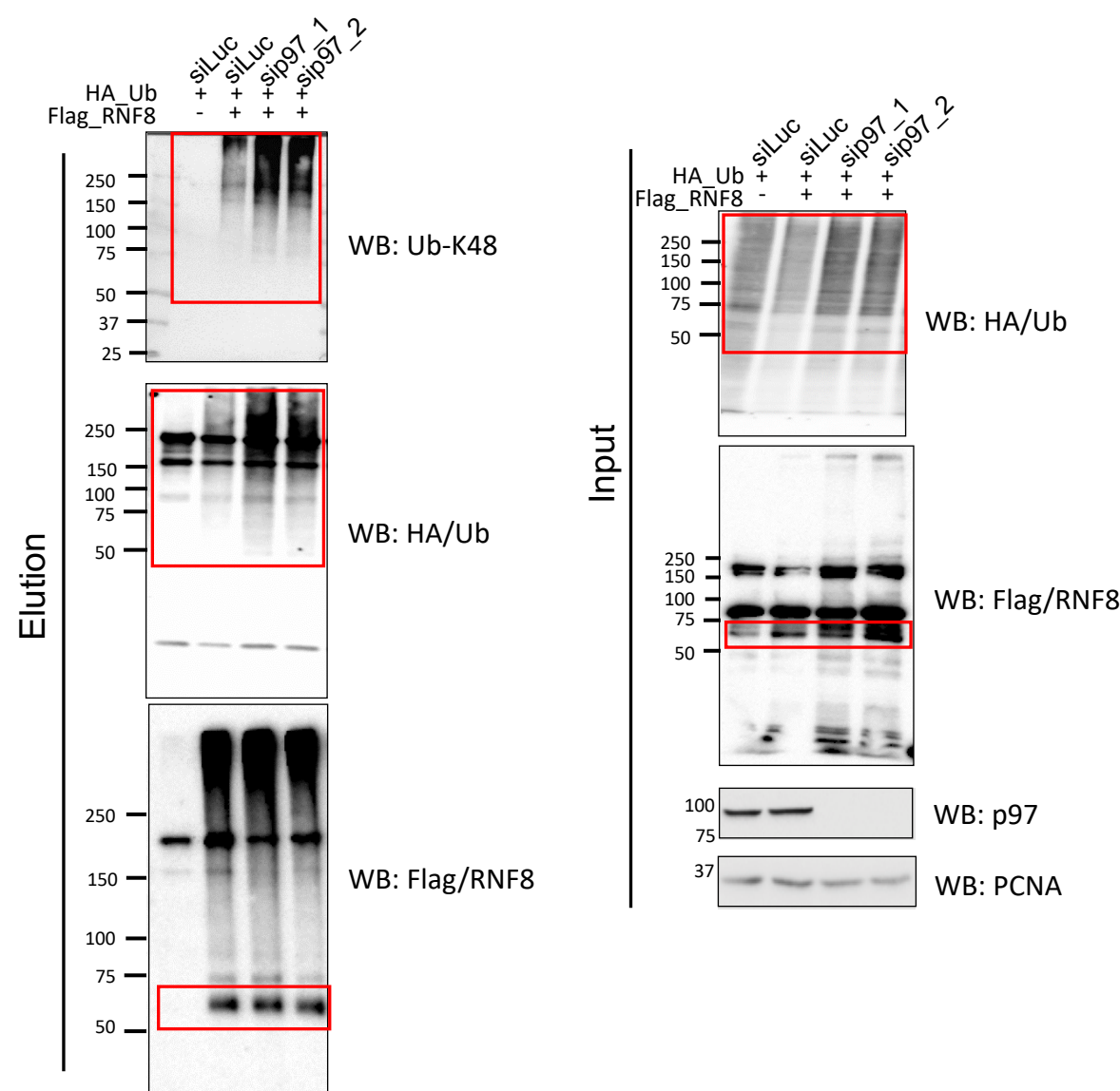

Figure 1K

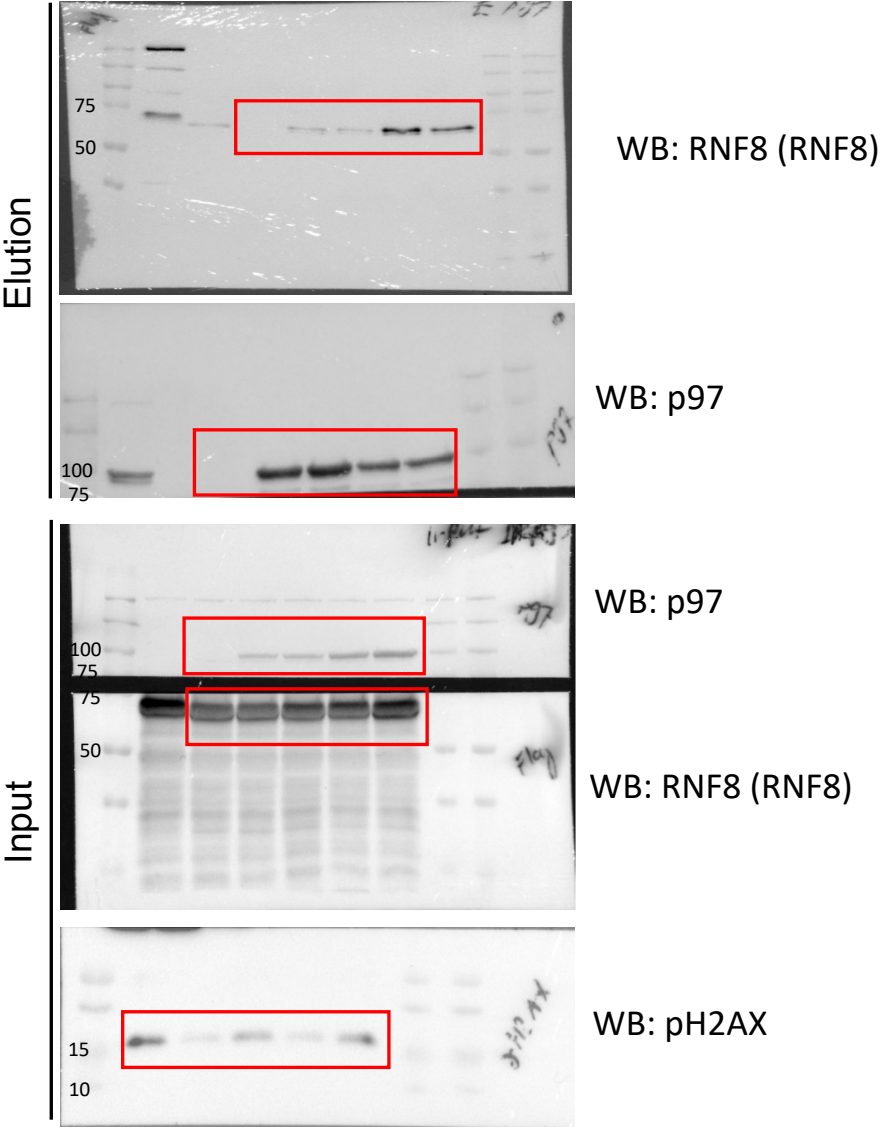

Supplement: Supplementary file 6 — Source Data for Figure 1 [file EMBJ-38-e102361-s004.pdf]

Figure 2A

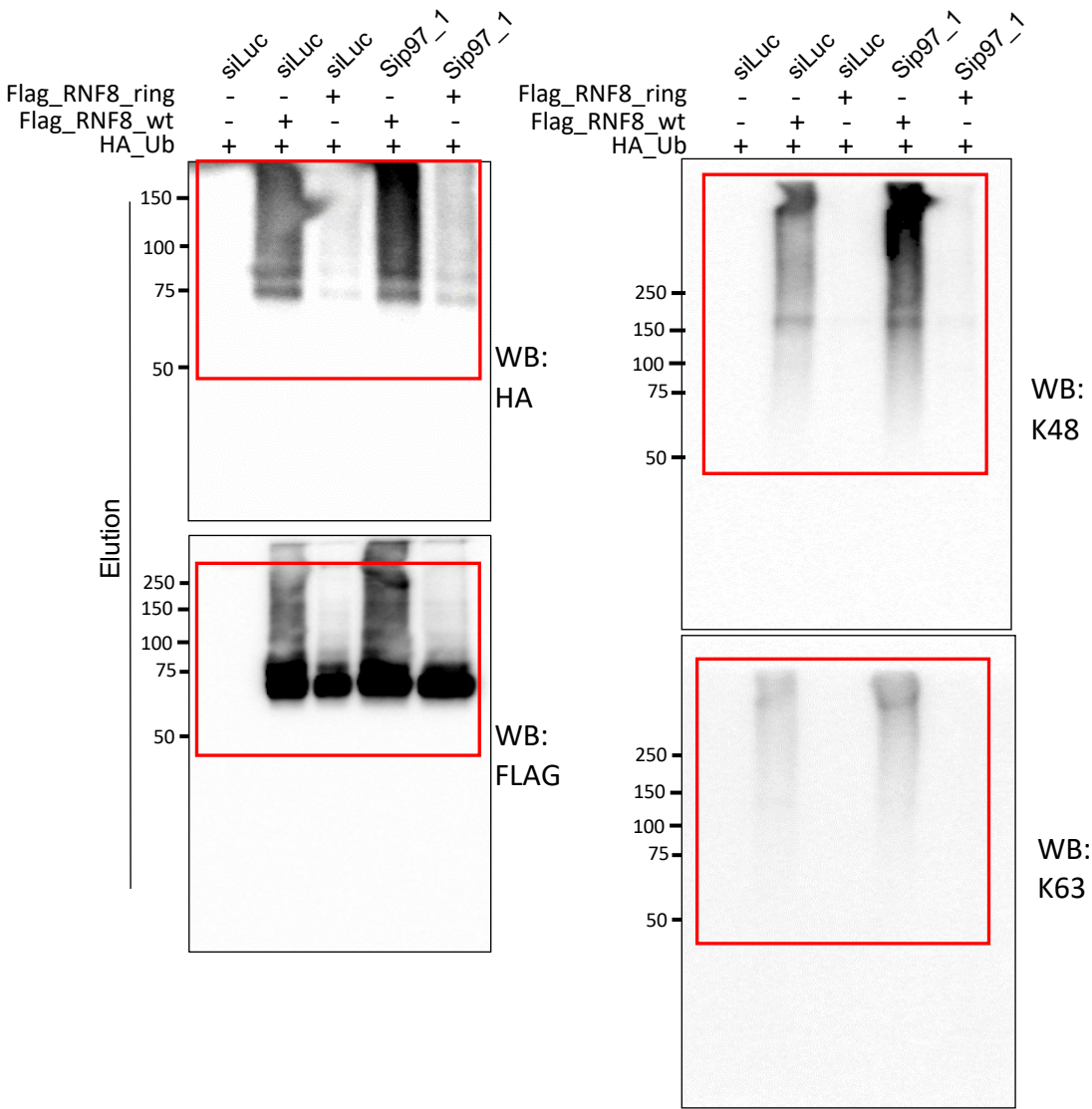

Figure 2B

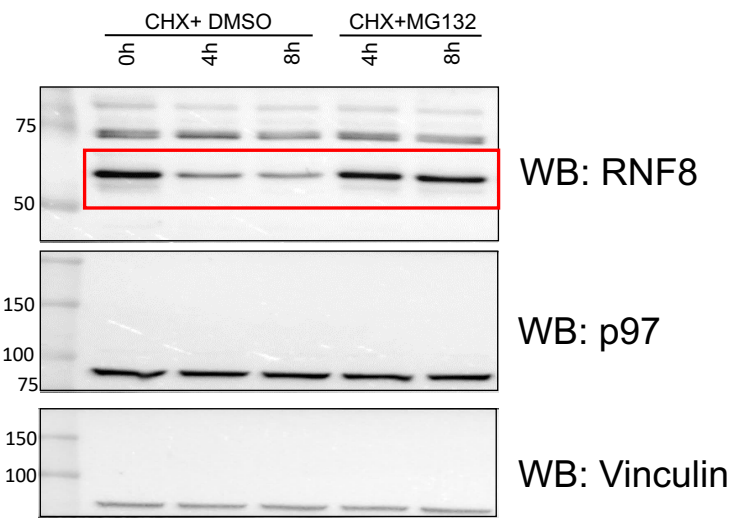

Figure 2D

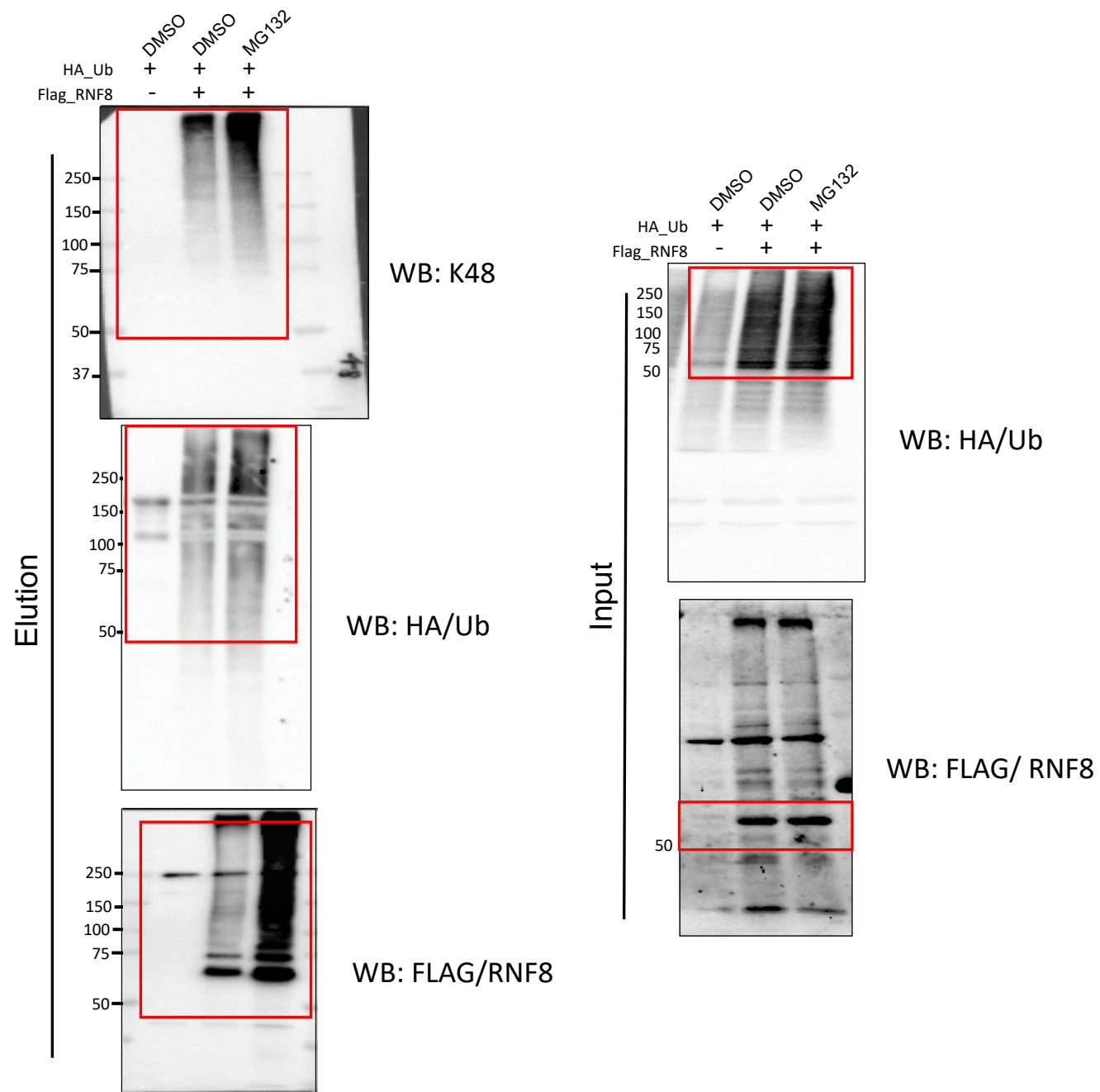

Figure 2E

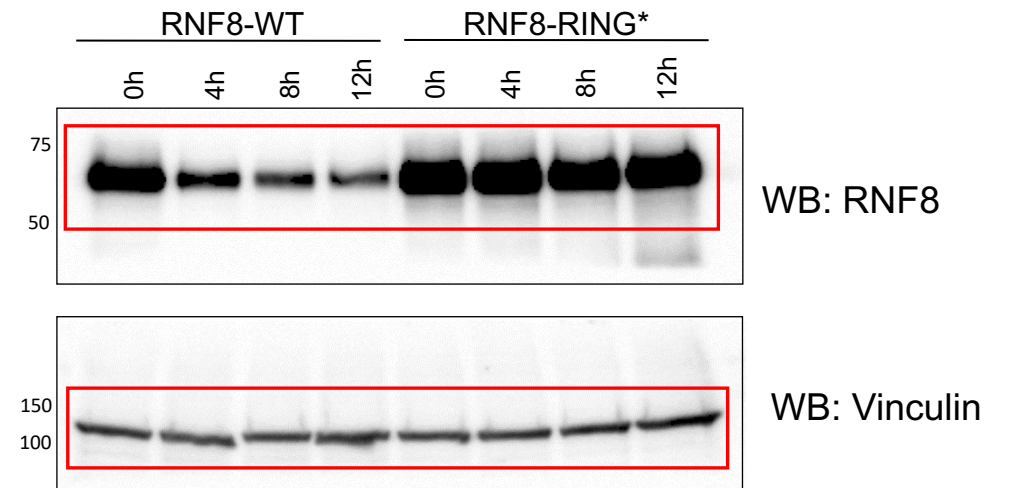

Supplement: Supplementary file 7 — Source Data for Figure 2 [file EMBJ-38-e102361-s005.pdf]

Figure 3C

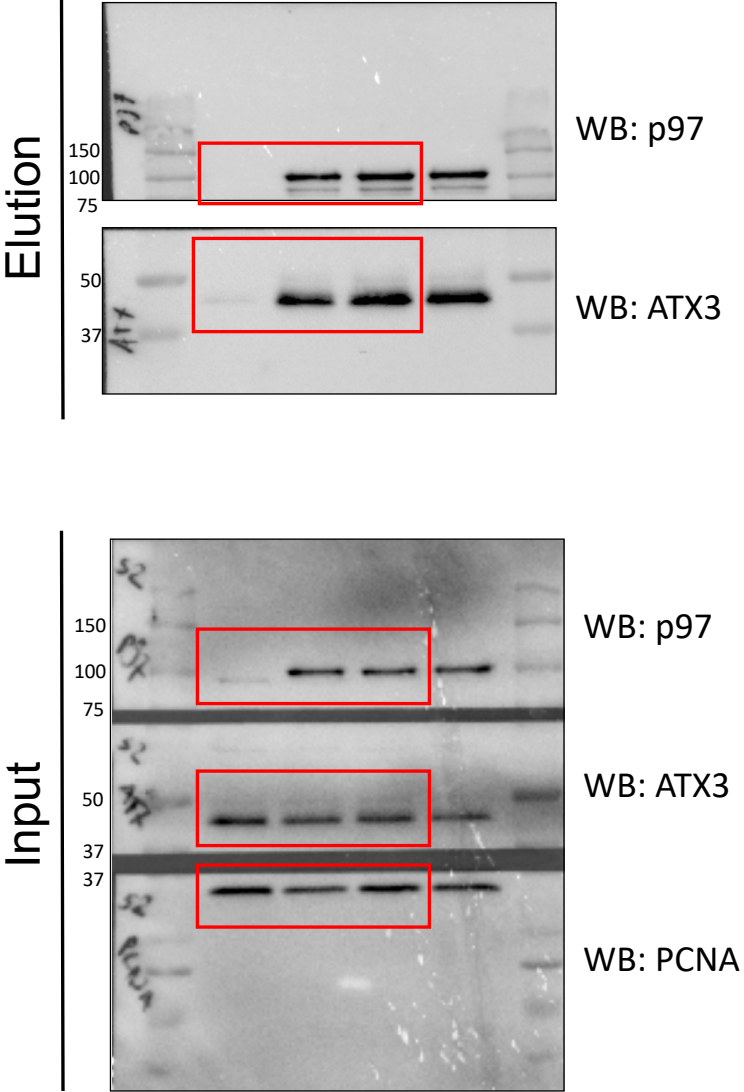

Figure 3D

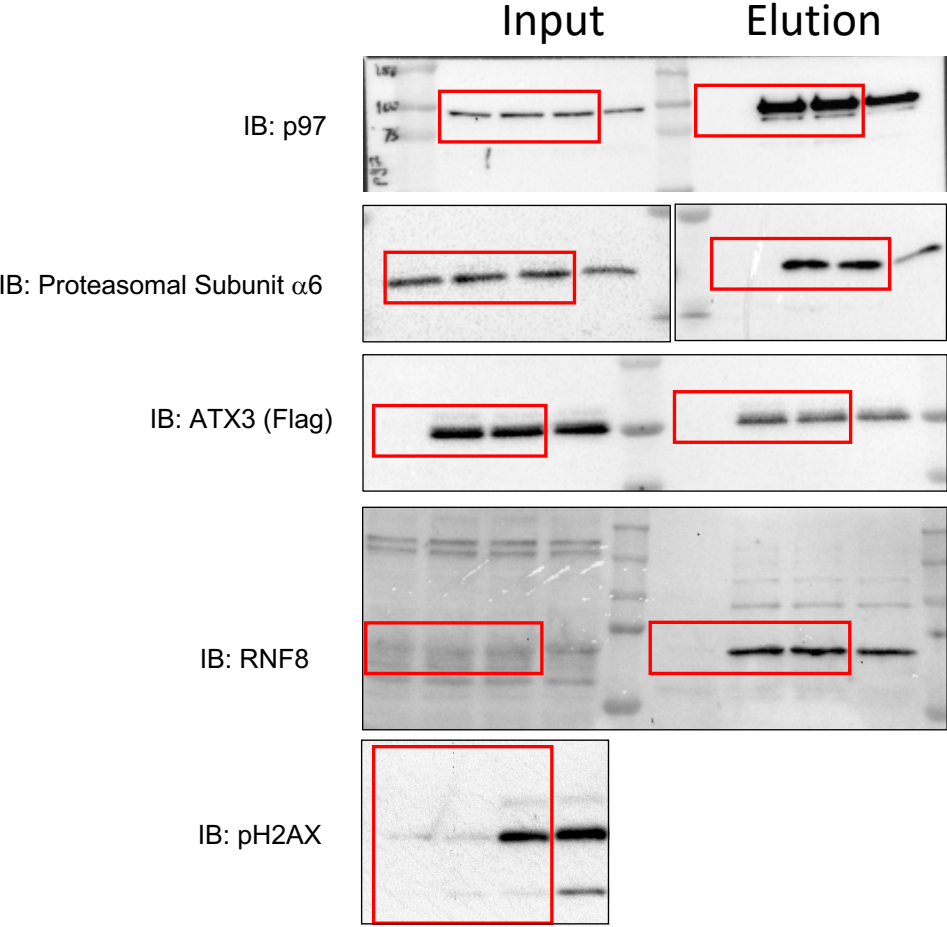

Figure 3E

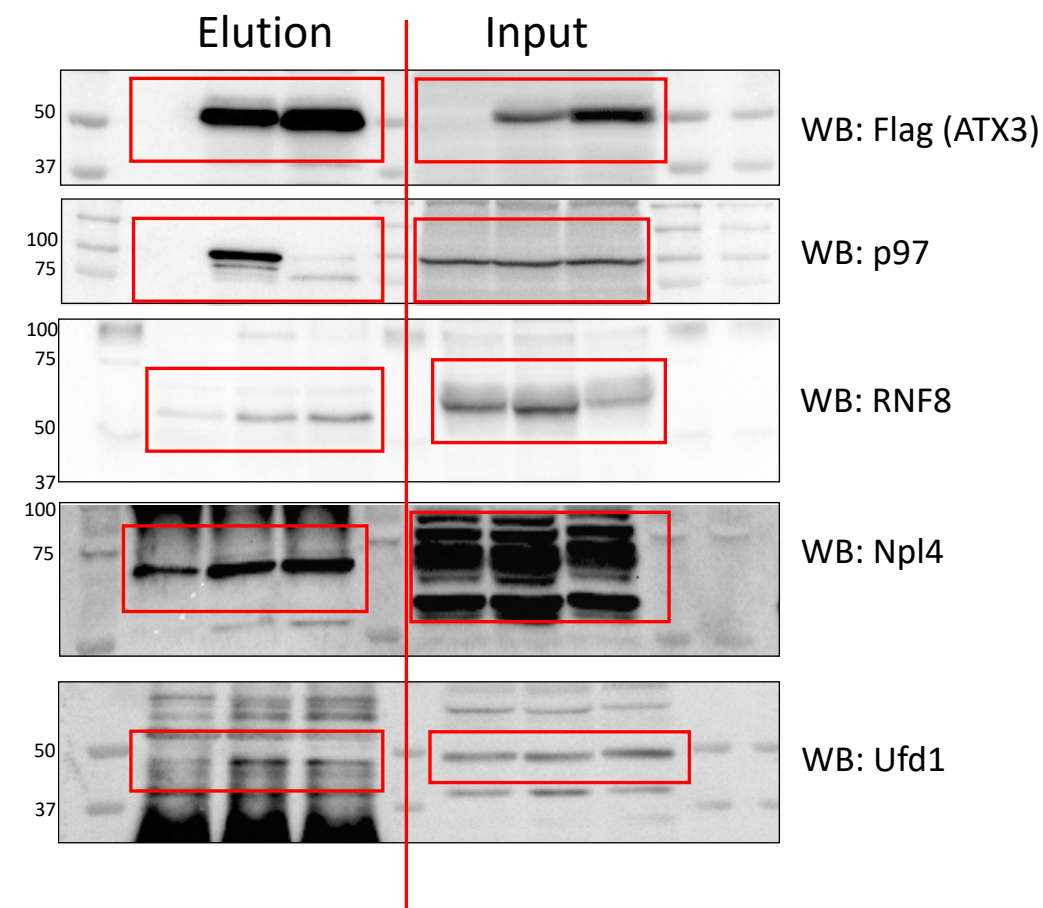

Figure 3F

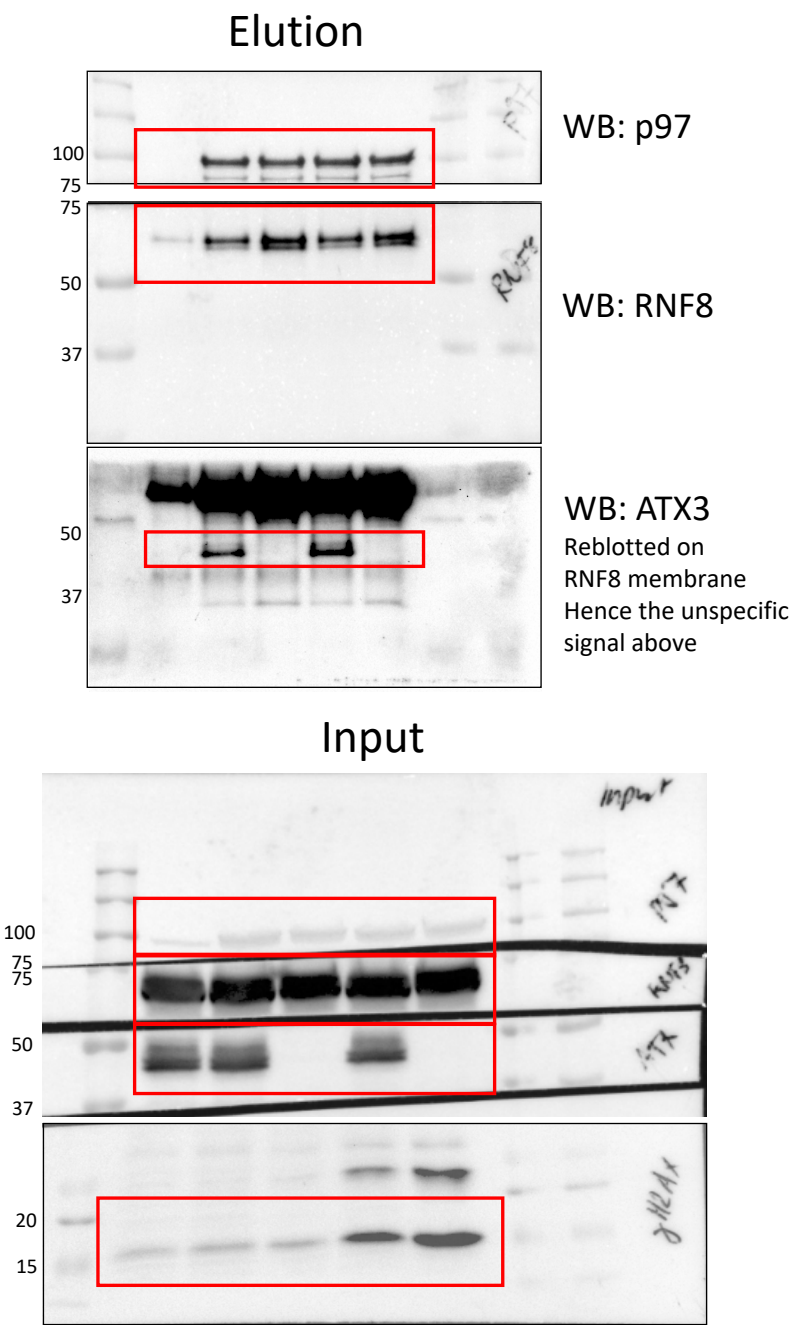

Supplement: Supplementary file 8 — Source Data for Figure 3 [file EMBJ-38-e102361-s006.pdf]

Figure 4A

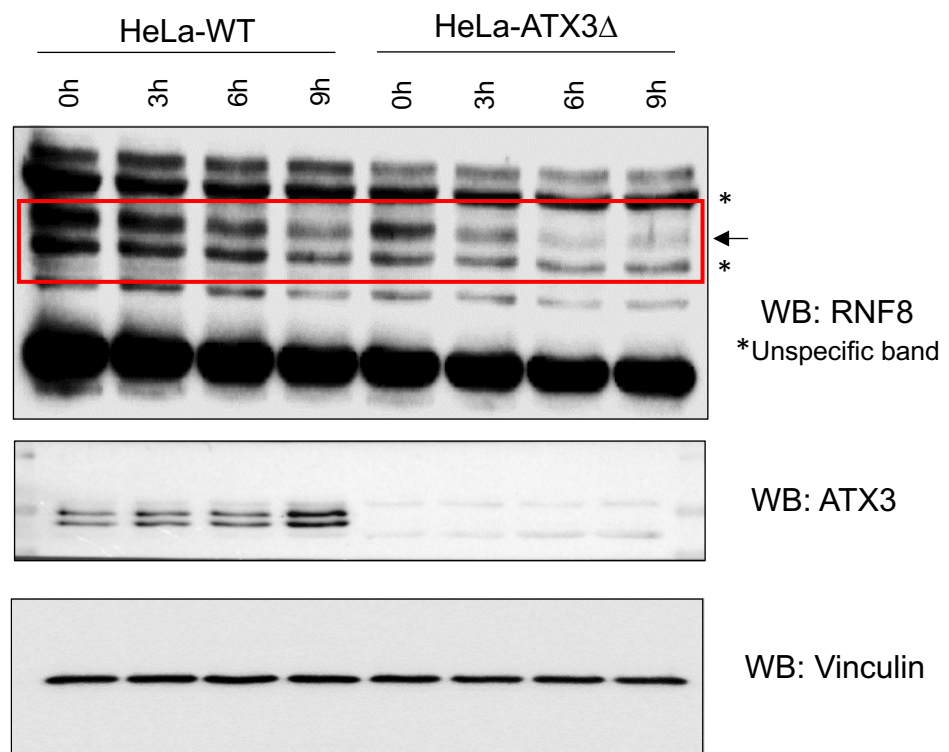

Figure 4C

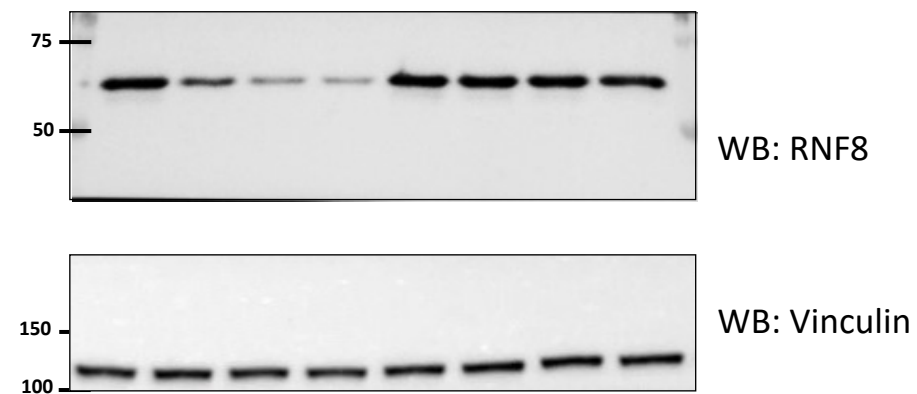

Figure 4D

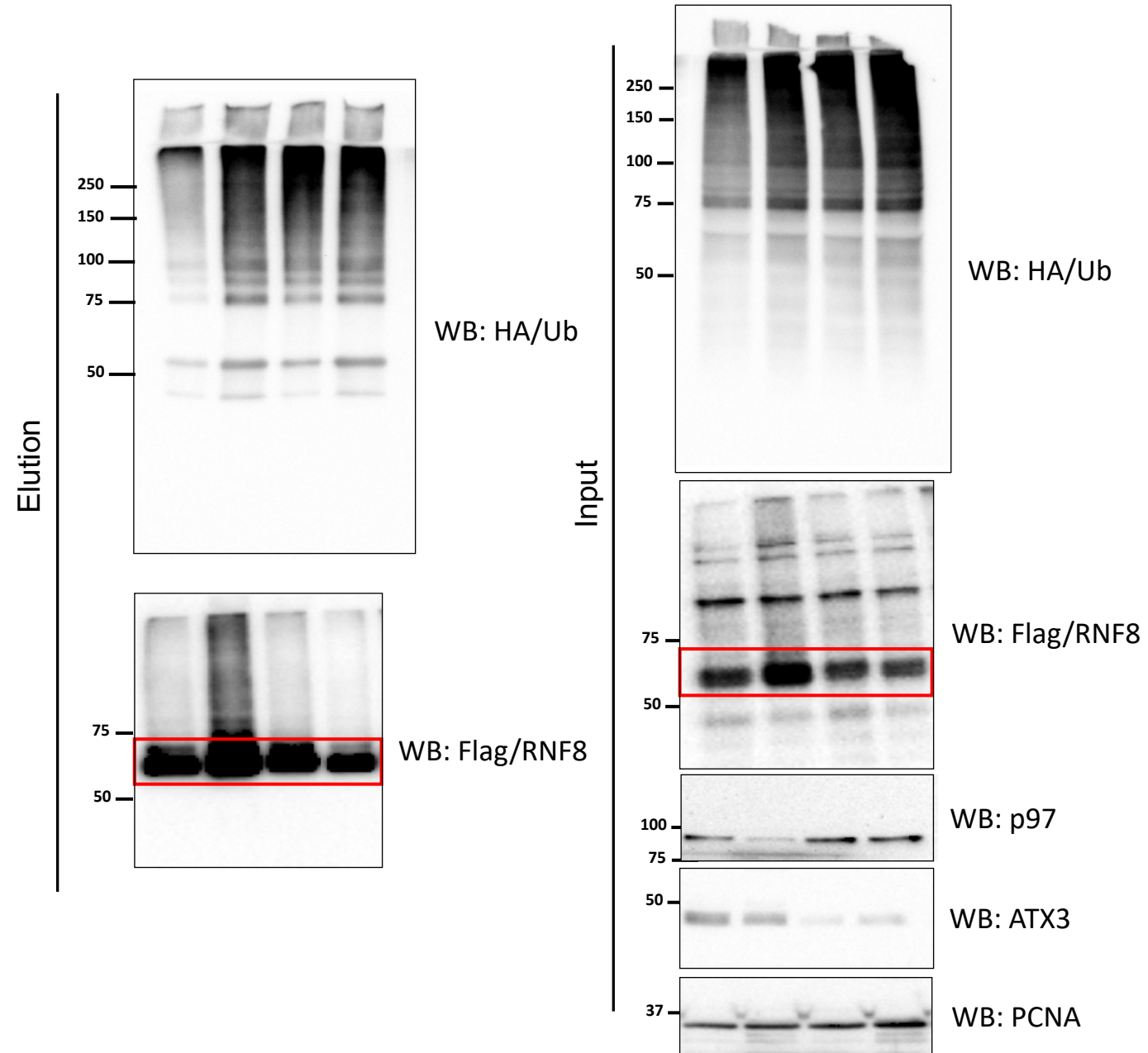

Figure 4E

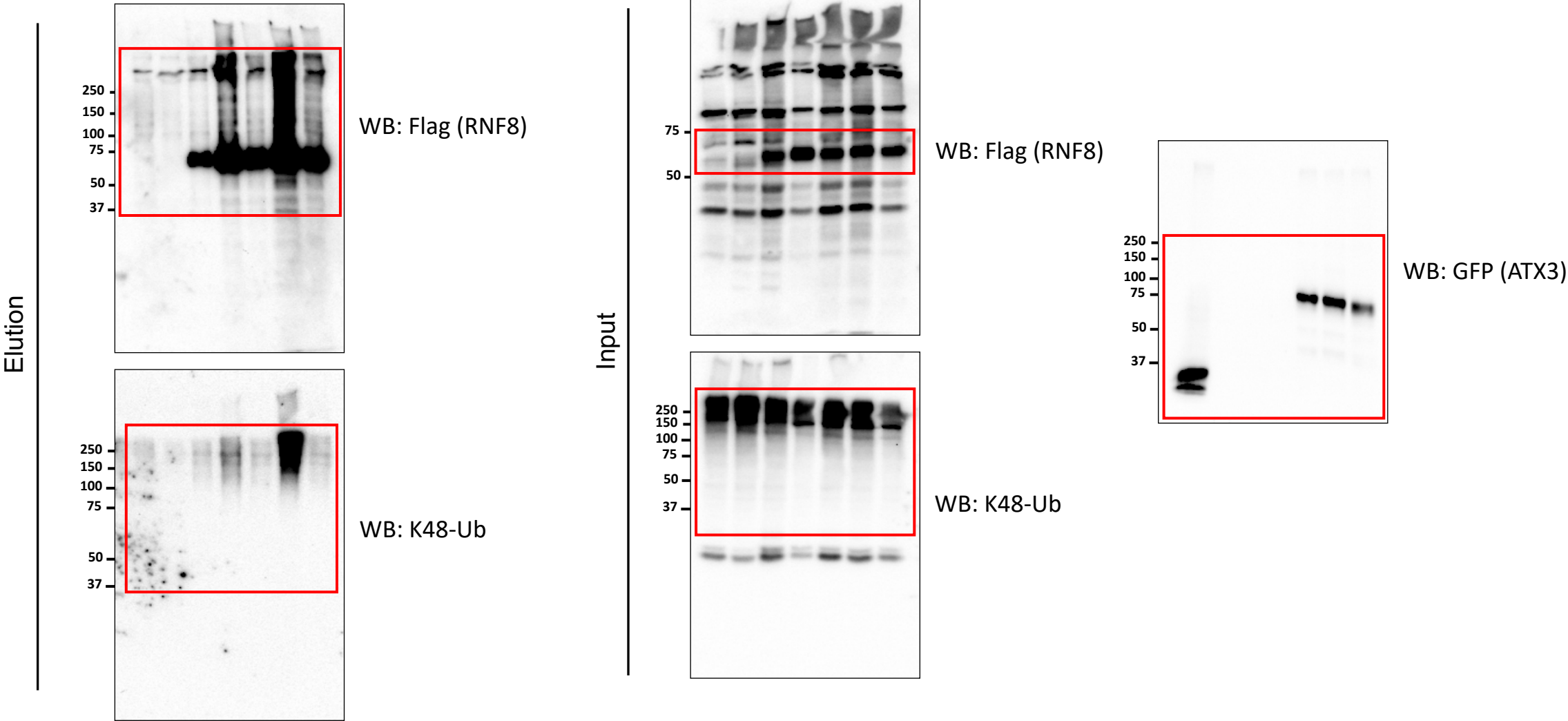

Supplement: Supplementary file 9 — Source Data for Figure 4 [file EMBJ-38-e102361-s007.pdf]

Figure 5A

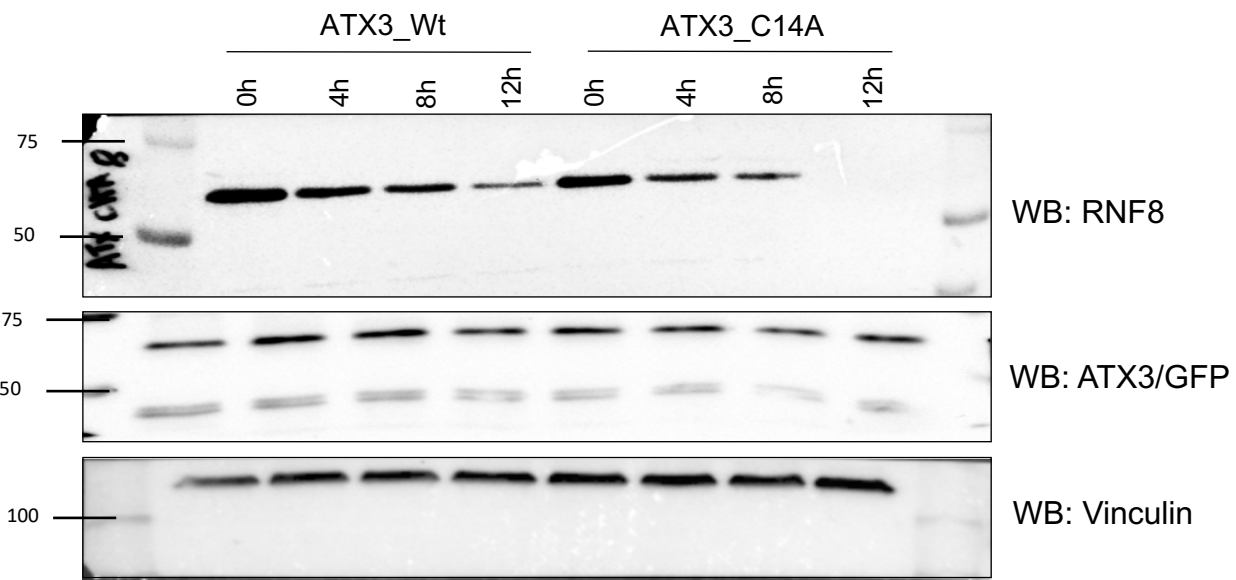

Figure 5C

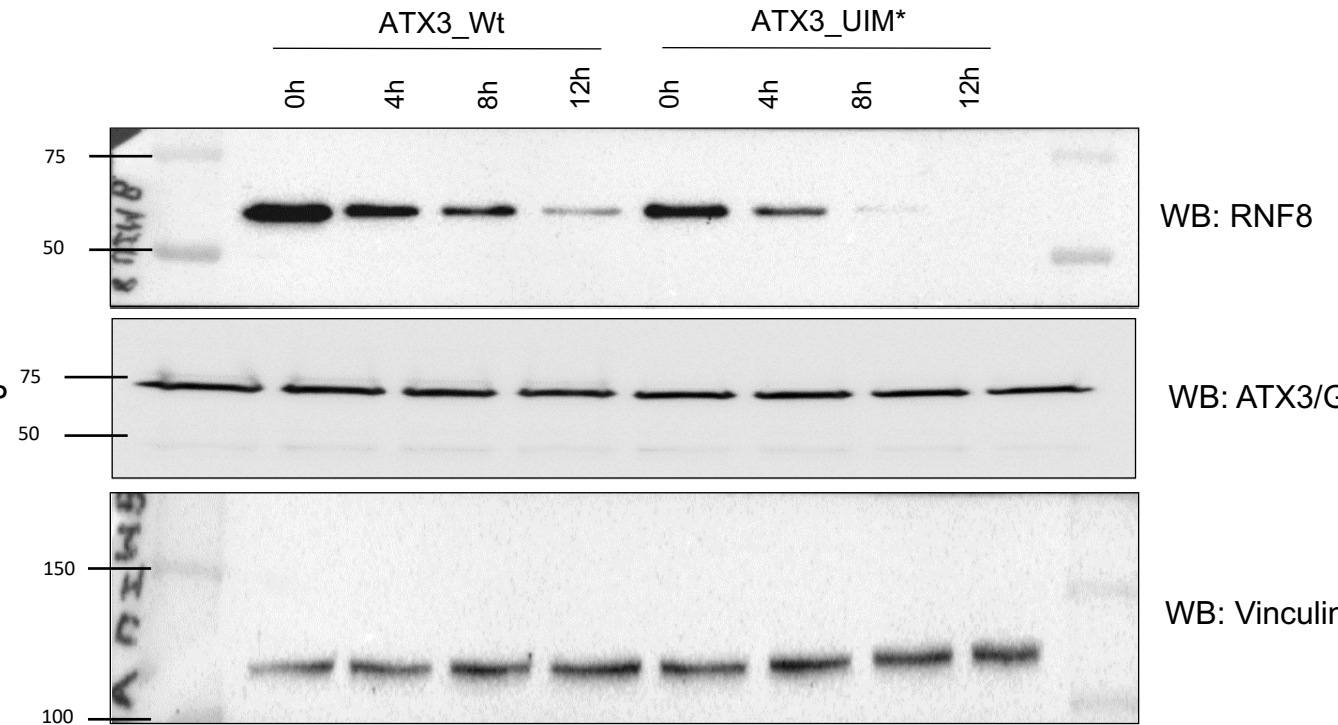

Figure 5E

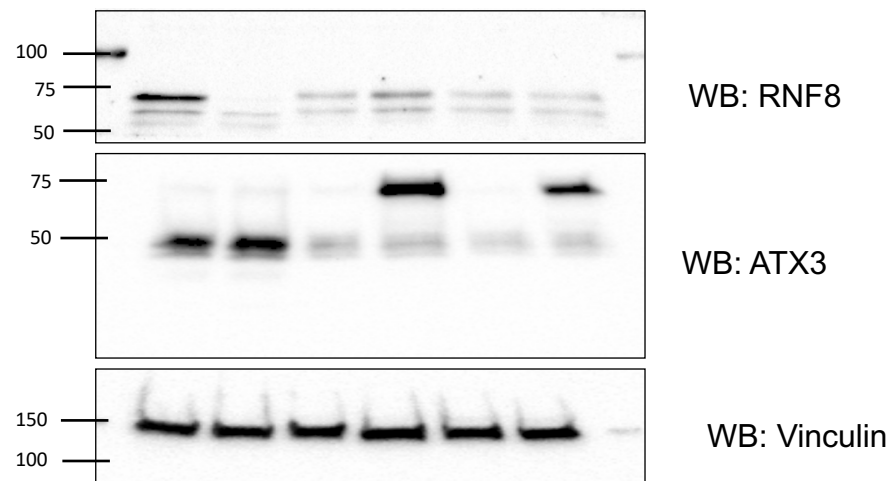

Supplement: Supplementary file 10 — Source Data for Figure 5 [file EMBJ-38-e102361-s008.pdf]

Figure 7E

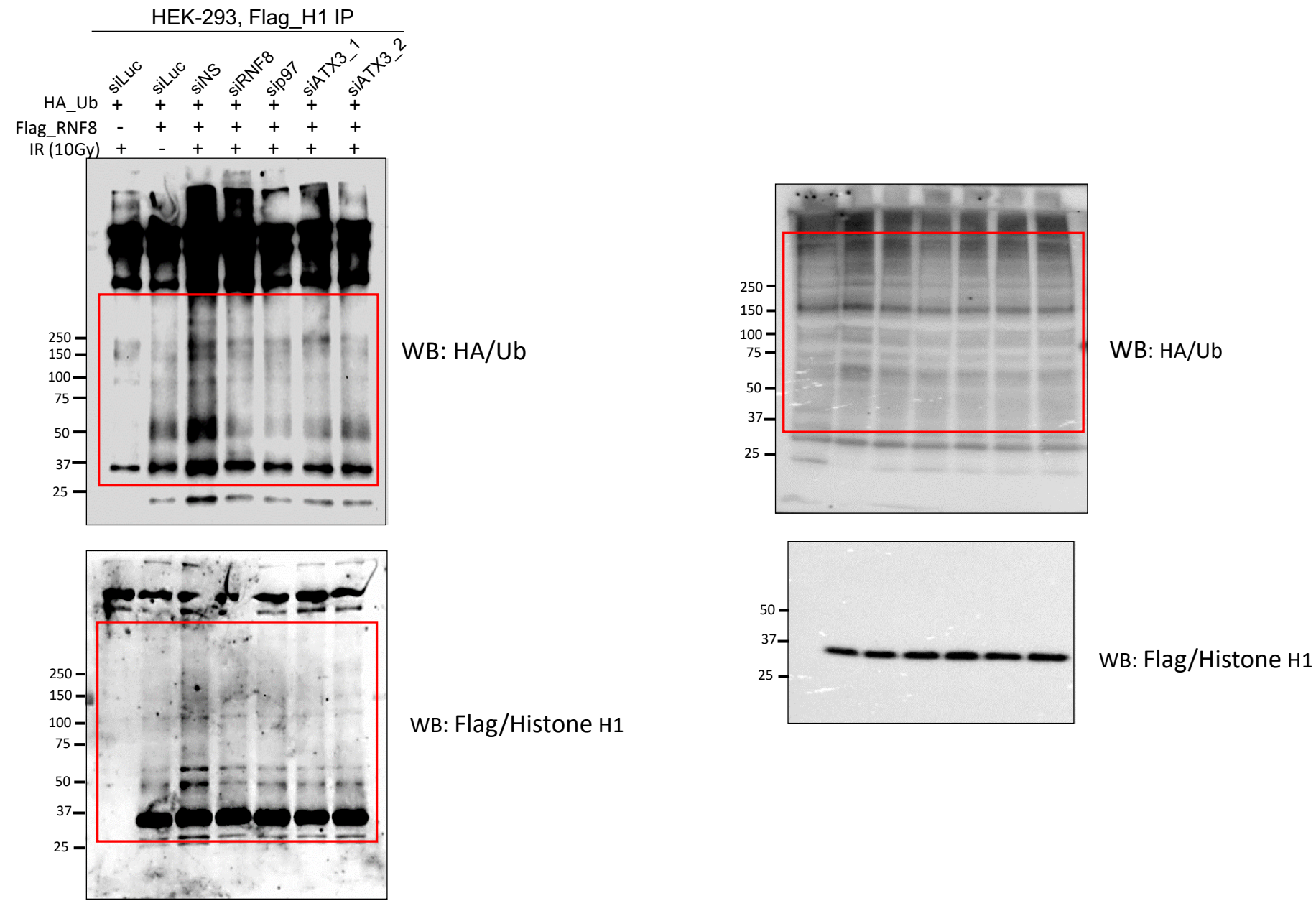

Supplement: Supplementary file 11 — Source Data for Figure 7 [file EMBJ-38-e102361-s009.pdf]

Figure 8C

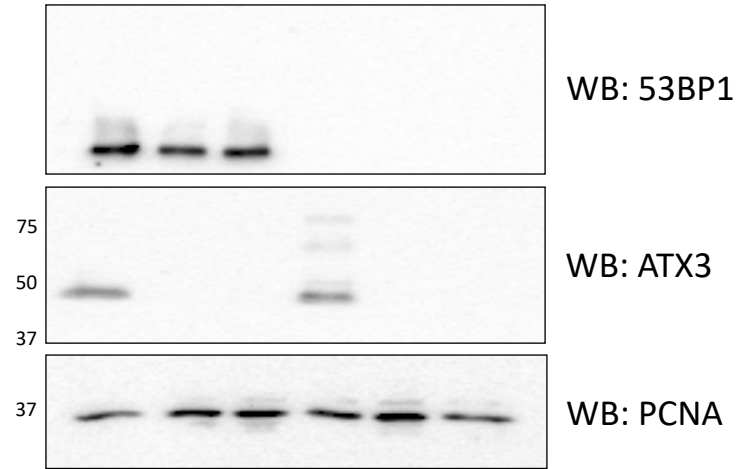

Figure 8D

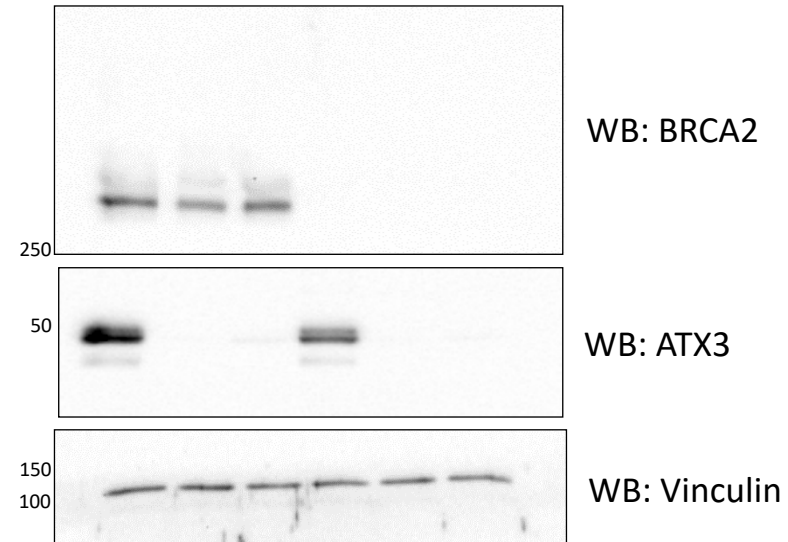

Figure 8G

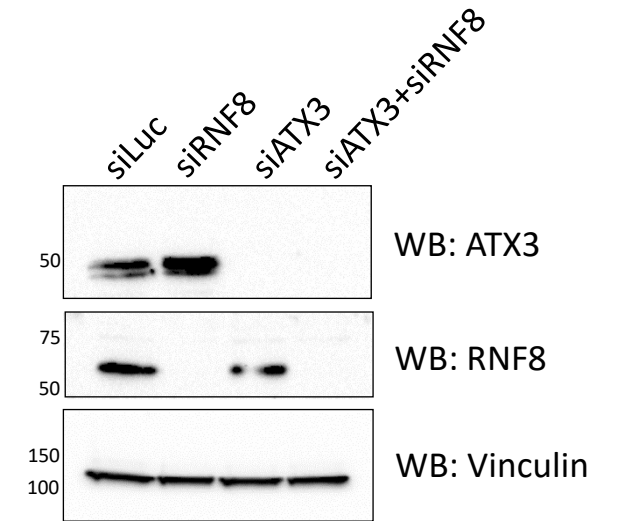

Supplement: Supplementary file 12 — Source Data for Figure 8 [file EMBJ-38-e102361-s010.pdf]
